# *Coe* Genes Are Expressed in Differentiating Neurons in the Central Nervous System of Protostomes

**DOI:** 10.1371/journal.pone.0021213

**Published:** 2011-06-17

**Authors:** Adrien Demilly, Elena Simionato, David Ohayon, Pierre Kerner, Alain Garcès, Michel Vervoort

**Affiliations:** 1 University of Paris Diderot, Sorbonne Paris Cité, Institut Jacques Monod, UMR 7592 CNRS, Paris, France; 2 Institut des Neurosciences de Montpellier, INSERM U-1051, Montpellier, France; 3 Institut Universitaire de France, Paris, France; MRC, University College of London, United Kingdom

## Abstract

Genes of the *coe* (*collier*/*olfactory*/*early B-cell factor*) family encode Helix-Loop-Helix transcription factors that are widely conserved in metazoans and involved in many developmental processes, neurogenesis in particular. Whereas their functions during vertebrate neural tube formation have been well documented, very little is known about their expression and role during central nervous system (CNS) development in protostomes. Here we characterized the CNS expression of *coe* genes in the insect *Drosophila melanogaster* and the polychaete annelid *Platynereis dumerilii*, which belong to different subgroups of protostomes and show strikingly different modes of development. In the *Drosophila* ventral nerve cord, we found that the Collier-expressing cells form a subpopulation of interneurons with diverse molecular identities and neurotransmitter phenotypes. We also demonstrate that *collier* is required for the proper differentiation of some interneurons belonging to the Eve-Lateral cluster. In *Platynereis dumerilii*, we cloned a single *coe* gene, *Pdu-coe*, and found that it is exclusively expressed in post mitotic neural cells. Using an original technique of *in silico* 3D registration, we show that *Pdu-coe* is co-expressed with many different neuronal markers and therefore that, like in *Drosophila*, its expression defines a heterogeneous population of neurons with diverse molecular identities. Our detailed characterization and comparison of *coe* gene expression in the CNS of two distantly-related protostomes suggest conserved roles of *coe* genes in neuronal differentiation in this clade. As similar roles have also been observed in vertebrates, this function was probably already established in the last common ancestor of all bilaterians.

## Introduction

The generation of neurons and glial cells is a complex and multi-step process, on which rely both the architecture and the activity of the bilaterian central nervous system (CNS). From the undifferentiated progenitors of the neural epithelium to the fully functional CNS cells, a large set of transcription factors and their combinatorial expression are required to control the different steps of commitment, patterning and differentiation. Among these transcription factors, collier/olfactory/early B-cell factor (COE) proteins form a specific family of helix-loop-helix (HLH) proteins, characterized by the presence of three highly conserved domains: a unique DNA binding domain (DBD); an immunoglobulin / plexin / transcription (IPT) domain putatively involved in both DNA/protein and protein/protein interactions; and an atypical HLH dimerization motif [1], [2], [3]. *coe* genes have been identified in the three major bilaterian lineages (deuterostomes and the two groups of protostomes, Ecdysozoa and Lophotrochozoa), as well as in the cnidarian *Nematostella vectensis*, the ctenophora *Mnemiopsis leidyi*, and the sponge *Amphimedon queenslandica* [3], [4], [5], [6], but not in plants or fungi, indicating that these genes form a metazoan specific family which would have arisen during early animal evolution. Members of the *coe* family are present as single genes in most studied species except for vertebrates which possess up to 4 homologs [3], [5], probably due to the two rounds of genome duplication which occurred during early vertebrate evolution [7].

Ebf1 and Olf-1, the two first identified members of the family, were originally described as a mouse transcriptional regulator of early B-cell differentiation [8], and a rat nuclear protein regulating the expression of the olfactory marker protein OMP [9], respectively. Several orthologs were subsequently identified in various vertebrate species, such as mouse, chicken, *Xenopus*, and zebrafish, and have been named either *Ebf* or *coe* [10], [11], [12], [13], [14]. A common theme for all the *coe* genes is a predominant expression in the developing nervous system, mostly in differentiating post-mitotic neural cells - an expression is nevertheless also found in non neural tissues such as the adult mouse spleen and the developing limb buds. In the mouse, *Ebf1*, *Ebf2* and *Ebf3* expression is detected in both peripheral nervous system (PNS) cells (olfactory sensory neurons, facial brachiomotor neurons and their respective precursors) and differentiating post-mitotic neurons of the CNS [15]. This latter profile is shared by all their orthologs in other vertebrates, except the zebrafish *Zcoe2* gene which is also expressed in progenitors of the primary neurons of the spinal cord, prior to the proneural gene *neurogenin* [11]. Extensive studies of vertebrate *coe* genes function have indicated their requirement at several crucial steps of neurogenesis, such as the coupling of cell cycle exit and migration from the subventricular proliferative zone toward the mantle layer of the neural tube, and proper neuronal differentiation [14], [16]. *Ebf1* is also required for axonal guidance of thalamic neurons projections, possibly by establishing the molecular patterning of the basal ganglia area [16], [17]. A *coe/ebf* gene isolated in a close-relative of vertebrates, the amphioxus *Branchiostoma floridae*, is expressed in many cells of the developing CNS, as well as in lateral isolated cells which may correspond to epidermal sensory neurons [18]. An expression associated to the developing nervous system is also found in the echinoderm *Strongylocentrotus purpuratus* [6], thus suggesting conserved functions for *coe* genes among deuterostomes.

In *Drosophila*, a single *coe* gene, *collier* (*col*), has been identified and shown to be required for the formation of the head/trunk intercalary segment [19], [20]. It has later been extensively studied for its involvement in several other developmental processes, such as the control of embryonic muscle identities [21], [22], wing patterning [23], [24], and differentiation of immune cells in response to parasitization [25], [26]. In addition, *col* is also expressed in the developing nervous system, both in cells of the ventral nerve cord (VNC) and in a few PNS neurons, a subset of the so-called multidendritic neurons, where it has been shown to control the elaboration of the characteristic dendritic arborization [27], [28], [29]. In the CNS, it specifies the identity of the peptidergic dorsal *Apterous* expressing (dAp) neurons, by triggering and participating to an original “feedforward cascade” of transcription factors at an early post-mitotic stage [30]. Despite this latter study, the identity of most of the cells expressing *col* in the VNC and its functions during CNS formation remain largely unknown. A *coe* gene, known as *unc-3*, has also been identified in another protostome, the nematode *C. elegans*, where it is involved in the axonal outgrowth of motor neurons [31] and in the specification of the identity of some chemosensory neurons [32]. Recently, an expression of *coe* genes in the developing nervous system has also been found in three other protostomes, a mollusc and two annelids [6].

In this article, we characterized the expression pattern of *coe* genes in the CNS of *Drosophila* and the annelid worm *Platynereis dumerilii*, species which belong to two different branches of the protostomes, Ecdysozoa and Lophotrochozoa respectively. In addition *Platynereis* displays vertebrate-like mechanisms ensuring CNS formation and patterning with key differences when compared to those found in *Drosophila* [33], [34], [35]. We first show that Col is expressed in about 10 to 15% of all *Drosophila* embryonic CNS neurons, corresponding exclusively to interneurons, as seen by co-expression with markers of a wide variety of interneuron types, while it is excluded from all known motor neurons. We also demonstrate that *col* is required for the proper differentiation of one population of interneurons, the *even-skipped* (*eve*)-lateral (EL) neurons. We then report the isolation of a single *coe* gene in *Platynereis* (referred to as *Pdu-coe*) and show that it is exclusively expressed in the nervous system (VNC, brain and PNS). In the VNC, *Pdu-coe* is only expressed in post-mitotic cells from which arise various neuronal subtypes. Taken together, our data confirm the remarkable conservation of COE protein structure among metazoans, as well as their predominant expression in the nervous system, almost exclusively in post-mitotic cells. However, striking differences are observed in the other expression domains of *coe* genes in the different bilaterian lineages [6], suggesting a plasticity of *coe* functions during evolution. We discuss a putative ancestral role for the *coe* gene family in neuronal differentiation in bilaterians, and hypothesize that a combinatorial mode of action involving interactions with several co-factors may explain the surprising discrepancy between high conservation of COE protein sequence and the plasticity of their exerted functions.

## Results

### Spatial and temporal analysis of *Drosophila* Col expression in the trunk CNS

**Figure 1:**
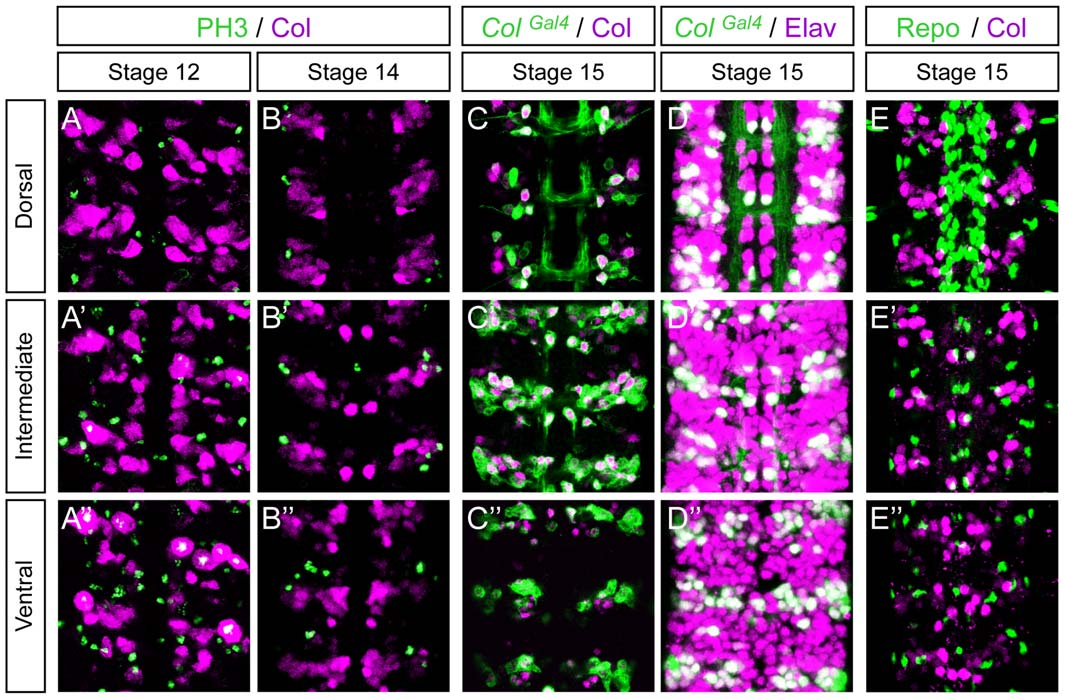
Col is expressed in neurons but not in glial cells in the embryonic *Drosophila* VNC. (A–A″) Col expression (magenta) relative to mitotic pattern as detected by Phospo-Histone H3 (PH3, green). At stage 12, two to three PH3/Col expressing cell are typically found per hemisegment in the ventral region of the VNC. (B–B″) At stage 14 expression of Col does not overlap with PH3 indicating that Col is only expressed in postmitotic cells. (C–C″) At stage 15 Col expression overlaps extensively with expression of *Col^Gal4^*. (D–D″) At stage 15 *Col^Gal4^* reveals that *Col* expressing cells are neurons (Elav+). (E–E″) At stage 15 no overlap between Col and Repo is found showing that Col is not expressed in glial cells. Dorsal, Intermediate and Ventral indicate the different focal planes of the VNC. In all panels three consecutive segments are shown. Anterior is upwards in all panels.

Previous studies showed an expression of the *Drosophila col* gene in the developing CNS [19], however without a detailed identification of the cells that express *col*, except for a few thoracic interneurons [30]. Here we used antibodies raised against Col [21], [30] and a *col-gal4* line [26] to further characterize its expression pattern in the trunk CNS. Col expression in the VNC was first detected at early stage 12 in about 20 cells per hemisegment and increased to approximately 50 cells per hemisegment at stage 14 and later (Figure 1A–A″ to C–C″ and Figure S1 B, B′). The position and size of these cells, as well as the timing of Col expression, suggest that they are postmitotic neural cells. To confirm this hypothesis, we performed double-labelings with antibodies against Col and the phosphorylated form of histone H3 (PH3) which reveals cells undergoing mitosis. In the ventral layer of the VNC of stage 12 embryos, we typically found two to three Col^+^ PH3^+^ cells per hemisegment probably ganglion mother cells (GMCs), but none at later stages (Figure 1A–A″, B–B″), indicating that Col is mainly (at stage 12) or only (at stage 14 and later) expressed in postmitotic neural cells.

To find out whether Col is expressed in neurons and/or glial cells, we performed double labelling with the neuronal marker Elav [36] and the glial marker Repo [37]. At stage 15, we found co-expression with Elav (Figure 1D–D″), but no overlap with Repo (Figure 1E–E″), indicating that Col is expressed in neurons but not in glial cells. Accordingly, in *col-gal4 / UAS-CD8-GFP* embryos, GFP decorates many neurites, confirming the neuronal expression of *col* (Figure S1). In addition, many VNC neurons retained *col* expression into larval stages (L2, see Figure S1; L1 and L3, not shown). We therefore conclude that *col* is mainly expressed in post-mitotic differentiating and differentiated neurons.

### *Drosophila* Col is expressed in various populations of interneurons, but not in motor neurons

**Figure 2:**
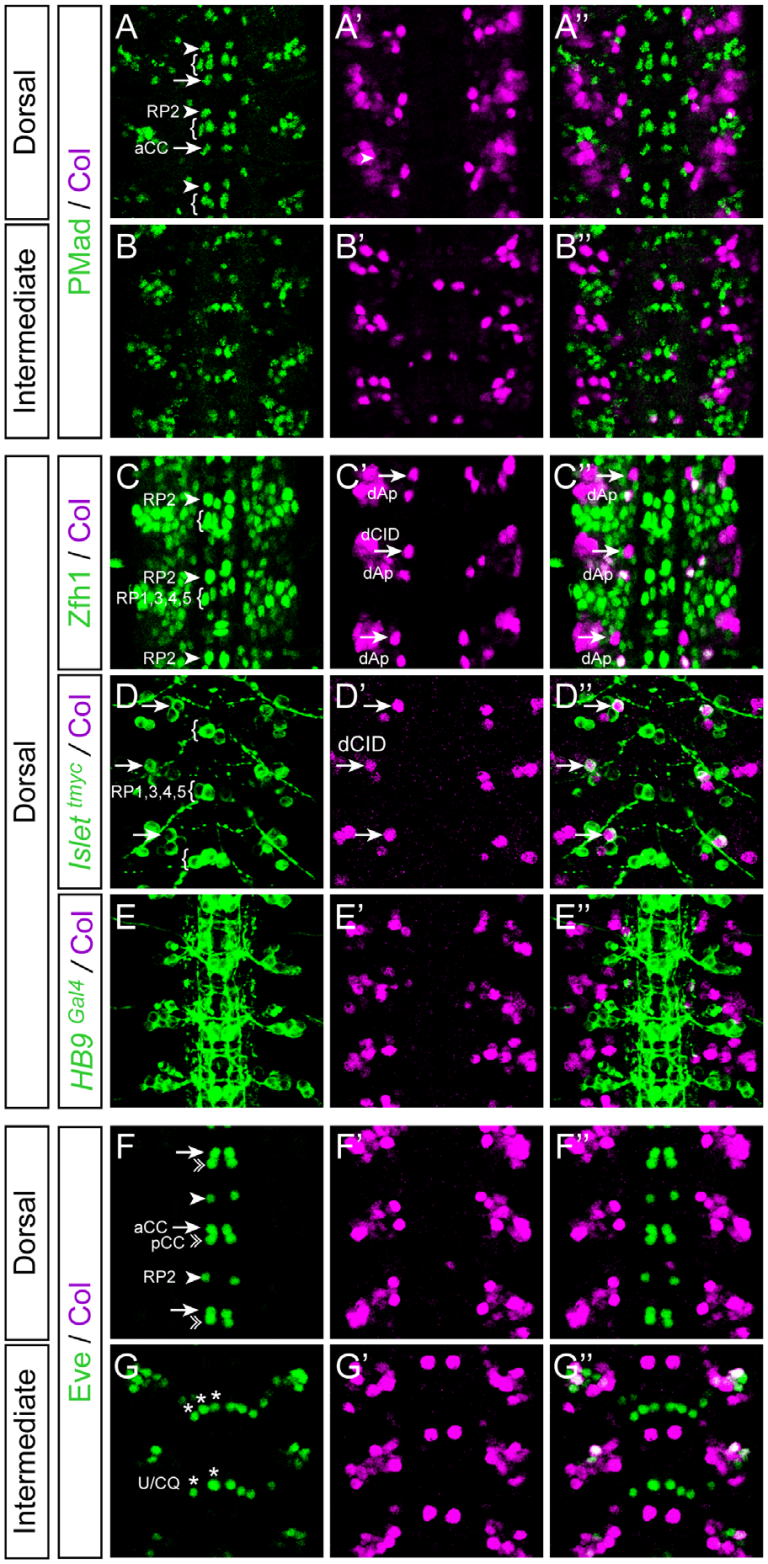
Col is not expressed in motor neurons. Stage 15 embryos stained with Col and different motor neuron markers. (A–B″) No overlap between Col and pMad is found in any of the different regions of the VNC examined. (C–C″) Col and Zfh1 expression only overlaps in the dorsal apterous neuron (dAp). (D–D″) Col is only found expressed in one *Islet-tau-mycEGFP*+ interneuron (see also Figure 3) that we have named dCID (dorsal Col, Islet, Dachshund) neuron and excluded from the identifiable Islet+ motor neurons RP1–5. (E–E″) Expression of Col and *HB9^Gal4^* is mutually exclusive. (F–G″) Col is not expressed by the Eve+ aCC, RP2 and U1–5 motor neurons neither by the pCC interneuron. Arrowheads: RP2 motor neuron; arrows: aCC motor neuron; arrows in (C–D″) indicate dCID interneuron; asterisks: U/CQ motor neurons (U1–5); brackets: RP1, 3, 4, 5 motor neurons; double arrowheads: pCC interneuron.

We next proceeded to the identification of the neurons expressing Col at stage 15 using a large set of markers widely covering the neuronal diversity of the *Drosophila* VNC. To find out whether Col is expressed in motor neurons, we first performed a double labelling using antibodies against Col and the phosphorylated form of Mad (pMad), a general motor neuron marker [38], [39], and found no-colocalization (Figure 2A–A″, B–B″). We confirmed this observation using several other motor neuron markers. Zfh1 is expressed in all somatic motor neurons as well as in one identified interneuron per hemisegment, the dAp neuron [39]. We found co-localization between Col and Zfh1 expression only in one dorsal neuron, much probably the dAp neuron (where Col expression has been previously characterized; [30]), but not in the Zfh1 expressing motor neurons (Figure 2 C–C″; Figure S2). Accordingly, Col is not expressed in the *islet1* ^+^ and *HB9* ^+^ RP1–3–4–5 motor neurons that innervate ventral muscles through the intersegmental nerve b (ISNb) [40], [41], nor in the Eve^+^ aCC, RP2 and U/CQ motor neurons innervating the dorsal muscles through the intersegmental nerve dorsal most (ISNdm) [42], [43], [44], nor in the *BarH1* ^+^ motor neurons innervating lateral muscles through the SNa nerve [45] (Figure 2 D–D″ to G–G″; Figure S2). Taken together, these observations show the absence of Col expression in motor neurons.

**Figure 3:**
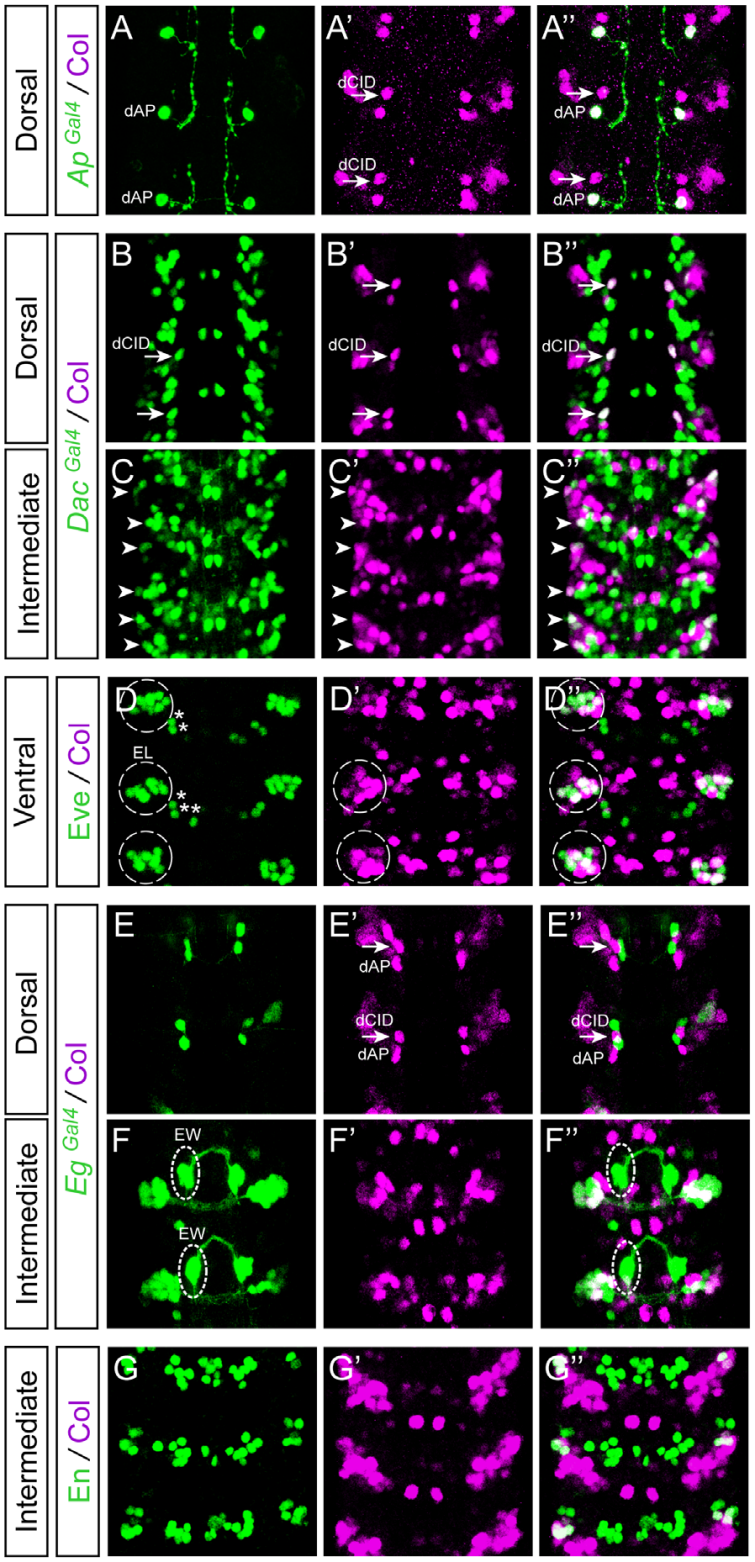
Col is expressed in discrete subsets of interneurons. Stage 15 embryos stained with Col and different interneuron markers. (A–A″) Col is expressed by the dAp (dorsal Apterous) interneuron (*Ap^Gal4^*+) and by the dCID interneuron (indicated by arrows). (B–B″) In the dorsal region of the VNC Col is only found expressed in one *Dachshund* (*Dac^Gal4^*) expressing interneuron that we have named dCID (dorsal Col, Islet, Dachshund) neuron (see also Figure 2). Note that the dCID is found at the vicinity of dAp. (C–C″) In the intermediate region of the VNC Col expression is found in 2 laterally located *Dac*+ interneurons indicated by the arrows. (D–D″) Overlap between Col and Eve is found in 4/5 EL (Eve Lateral) Eve+ interneurons. (E–E″) In the dorsal region of the VNC Col is not expressed with *Eagle* (*Eg^Gal4^*). Nevertheless note that the dAp and the dCID interneurons are located in a region where two *Eg*+ neurons are found. Note that here only two consecutive segments are shown. (F–F″) In the intermediate region of the VNC Col is not found expressed in the 3 *Eg* expressing serotoninergic neurons (EW1–3) but it is expressed in 3/4 *Eg*+ laterally located interneurons that belong the EL (Eve+) cluster. (G–G″) Col and Engrailed (En) expression only overlaps in 2 interneurons that are located in the lateral and intermediate region of the VNC.

Col is therefore expressed in some interneuron populations that we characterized further using several molecular markers. In the very dorsal part of the VNC, Col is reproducibly expressed in a pair of neurons in each hemisegment, one of which we suspected to be the Zfh1^+^ dAp neuron (Figure 2 C–C″). We confirmed this assumption by showing the co-expression of Col and *apterous* in this particular neuron (Figure 3A′–A″). The second neuron of the dorsal pair co-expresses Col and *islet* (Figure 2D–D″) as well as *dachshund* (*dac*) (Figure 3B–B″) which is exclusively expressed in interneurons in the VNC [46] - we therefore named this neuron the dCID neuron (dorsal Collier/Islet/Dac neuron). In the ventral part of the VNC, Col expression is detected in several interneurons, two to three of which also express *dac* (Figure 3C–C″). Four to five of the other Col^+^ neurons belong to the EL (Eve-lateral) cluster, which contains 9–10 neurons expressing Even-Skipped (Eve) [47] (Figure 3D–D″). EL neurons also express *eagle* [48] and we found a co-expression of Col and *eagle* in 4–5 EL neurons (Figure 3E–F″). We confirmed by a triple-labelling that some EL neurons express Eve, Col, and *eagle*. Finally, we found Col expressed in each hemisegment in 2 Engrailed^+^ interneurons [49] (Figure 3G–G″) and in 1–2 *Hb9^+^* interneurons (Figure S2). By contrast, no co-expression was found between Col and *dbx*, whose expression marks a large set of GABAergic interneurons [50] (Figure S3).

**Figure 4:**
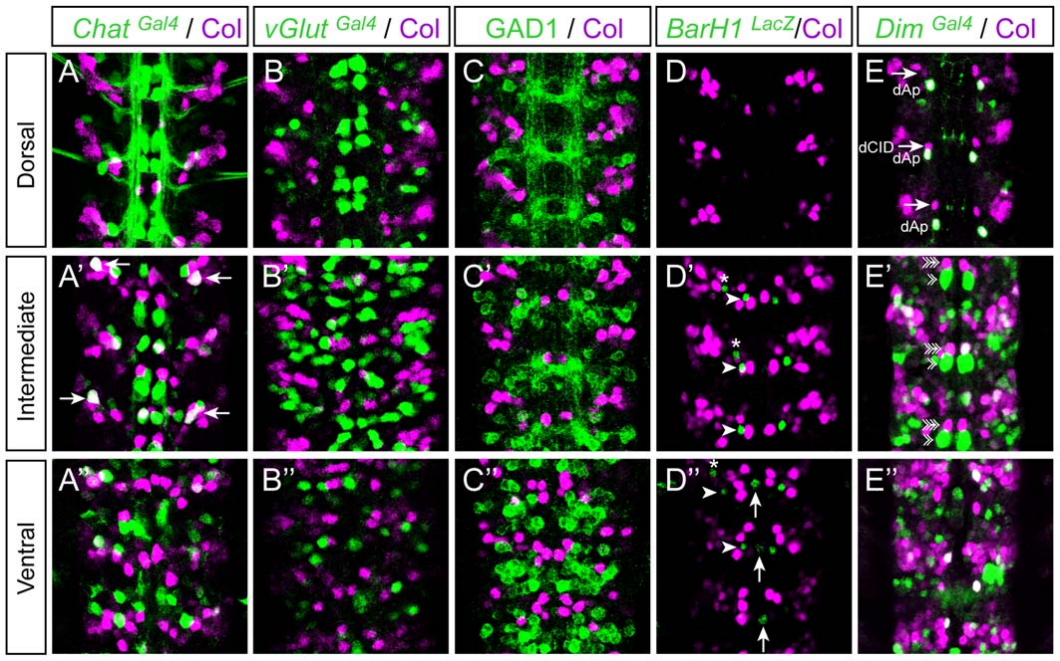
Col expression and neurotransmitter phenotypes in embryos. Late stage 16 embryos stained with Col together with *Chat^Gal4^* (Choline Acetyltransferase) (A–A″) and *vGlut^Gal4^* (vesicular Glutamate transporter) (B–B″). Stage 15 embryos stained with Col together with GAD1 (Glutamic Acid Decarboxylase) (C–C″), *BarH1^LacZ^* (a marker for intermediate and ventrally located dopaminergic interneurons) (D′, D″) and *Dim^Gal4^* (*dimmed*, a marker for a large class of peptidergic neurons) (E–E″). There is no co-labeling between Col and *vGluT*, nor Col and GAD1 and neither Col and *BarH1,* indicating that Col is not expressed in glutamatergic, gabaergic nor dopaminergic neurons. Only one neuron, located in the lateral and intermediate region of the VNC (arrows in A′), is found co-expressing Col and *Chat* suggesting that only a very discrete subpopulation of cholinergic interneurons express Col at this stage. Co-localization between *Dim* and Col is found in the dAp neuron dorsally (E) and in a few cells located in the intermediate and ventral regions of the VNC (E′, E″). In (D′, D″) arrows and arrowheads point, respectively, towards the ventral unpaired dopaminergic neuron and two other Tyrosine Hydroxylase-positive cells located more laterally. Asterisks indicate SNa motoneurons. In (E–E″) arrows, arrowheads and double arrowheads point, respectively towards dCID, dMP2 and vMP2 neurons.

**Figure 5:**
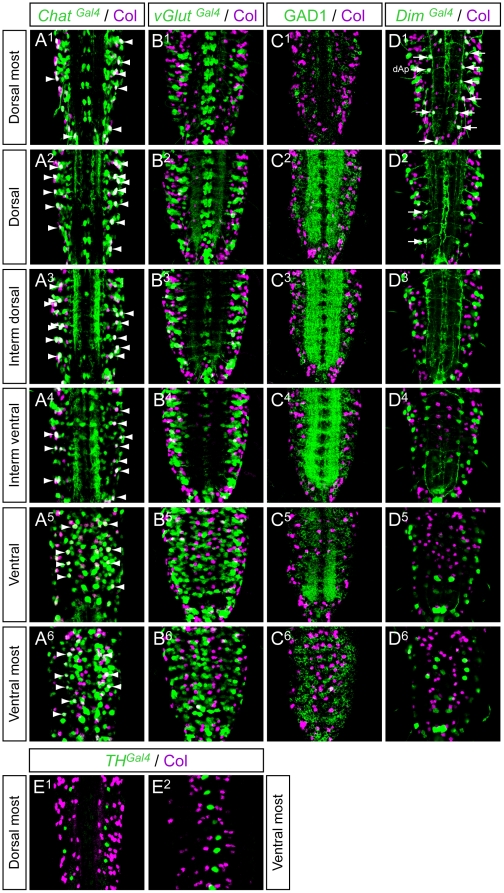
Col expression and neurotransmitter phenotypes in larvae. L1 larvae stained with Col together with *Chat^Gal4^* (A^1^–A^6^), *vGlut^Gal4^* (B^1^–B^6^), GAD1 (C^1^–C^6^), *Dim^Gal4^* (D^1^–D^6^) and *TH^Gal4^* (E^1^–E^2^). There is no co-labeling between Col and *vGluT*, nor Col and GAD1, nor Col and *TH,* indicating that Col is not expressed in glutamatergic, gabaergic nor dopaminergic neurons. Only the dorsally located dAp neuron is found co-expressing Col and *Dim* (arrows in D^1^) and a subset of Col expressing neurons are *Chat* positive (8 to 10 neurons per hemisegment; arrowheads in A^1^–A^6^).

We then looked for a possible correlation between Col expression and neurotransmitter phenotypes in the VNC. To define whether Col is expressed in glutamatergic, cholinergic, or GABAergic neurons, we performed double-labellings with Col and *vGlut^Gal4^ (vesicular Glutamate transporter)*, *ChAT* (*Choline Acetyltransferase*), and GAD1 (glutamic acid decarboxylase 1), respectively. In late stage 16 embryos Col and *Chat* were found to be co-expressed in only one neuron in each hemisegment (Figure 4A–A″) and we did not detect any co-expression of Col with *GluT* and Gad1 (Figure 4B–C″). In first instar larvae, a stage where neurons are fully differentiated (at least at the electrophysiological level), we found that 8 to 10 Col expressing neurons per hemisegment also express *Chat* (Figure 5A ^1^–A^6^) and thus are cholinergic while Col expression is excluded from neurons expressing either v*Glut* (Figure 5B ^1^–B^6^) or *Gad1* (Figure 5C ^1^–C^6^). Both in embryonic and larval stages we did not detect Col expression in the ventrally and dorsally located sets of dopaminergic neurons which express *BarH1* [45] and *Tyrosine Hydroxylase* (*TH*) [51] (Figure 4D–D″ and Figure 5E ^1^–E^2^), nor in the serotoninergic neurons (named EW) which express *eagle* [52] (Figure 3E–E″ and not shown). The *Drosophila* VNC also contains peptidergic neurons, most of which expressing the *dimmed* gene [53]. In late stage 16 co-expression of *dimmed* and Col is observed in 6–7 cells (Figure 4E–E″), indicating that only a small part of the Col-expressing neurons are peptidergic, and that most peptidergic neurons do not express Col at this stage. In L1 larvae only one dorsally located neuron, namely the dAp neuron, is found in each hemisegment to co-express Col and *dimmed* (Figure 5D ^1^–D^6^).

During the course of this analysis double labelling between Col and *dimmed* showed that Col is expressed, in each hemisegment, in a neuron immediately adjacent to one of the well-characterized *dimmed*-expressing neurons, dMP2 (Figure 4E′ arrowheads). These neurons are produced by very peculiar neuroblasts, named MP2, that divide only once to produce two neurons: dMP2 and vMP2. The first one extends its axon posteriorly in the CNS whereas the second one extends its axon anteriorly towards the brain and both act as pioneering neurons during embryogenesis [54]. In the most posterior segments, dMP2 neurons survive during larval stages, innervate the hindgut and produce insulin-like peptides [55]. The specification of dMP2 versus vMP2 identity involves the Notch signalling pathway and the Numb protein: during the division of MP2, Numb is partitioned into one of the daughters where it antagonizes Notch signalling, thus promoting the acquisition of the dMP2 fate – the other daughter cell which receives Notch signal becomes vMP2 [54], [56], [57] (Figure S4). Judging by their position, adjacent to dMP2, we were tempted to identify some of the Col expressing cells as vMP2 neurons (Figure 4E′ double arrowheads). To confirm this, we forced the expression of an activated form of Notch (Nintra) in the dMP2 neurons and in response found Col to be expressed in most of these neurons (Figure S4), thus indicating that, in the wild-type, Col is expressed in vMP2 under the control of Notch signalling.

All together our data indicate that Col is not expressed in motor neurons, but rather in several subpopulations of interneurons (together constituting 10 to 15% of all CNS neurons), some of which are well characterized. This includes the dAp peptidergic interneurons, part of the *eve*-lateral (EL) cluster, and the vMP2 interneurons in which *col* expression relies on Notch signalling. Moreover, neurons expressing Col do not produce glutamate, GABA, dopamine, nor serotonin, and only a subpopulation of them are peptidergic or cholinergic. Finally, we identified a previously uncharacterized interneuron, dCID, that can be recognized by its characteristic dorsal position in the close vicinity of dAp and by the expression of a unique combination of molecular markers (Col, *islet*, and *dac*).

### *col* is required for the proper differentiation of some Eve-expressing interneurons in the *Drosophila* VNC

**Figure 6:**
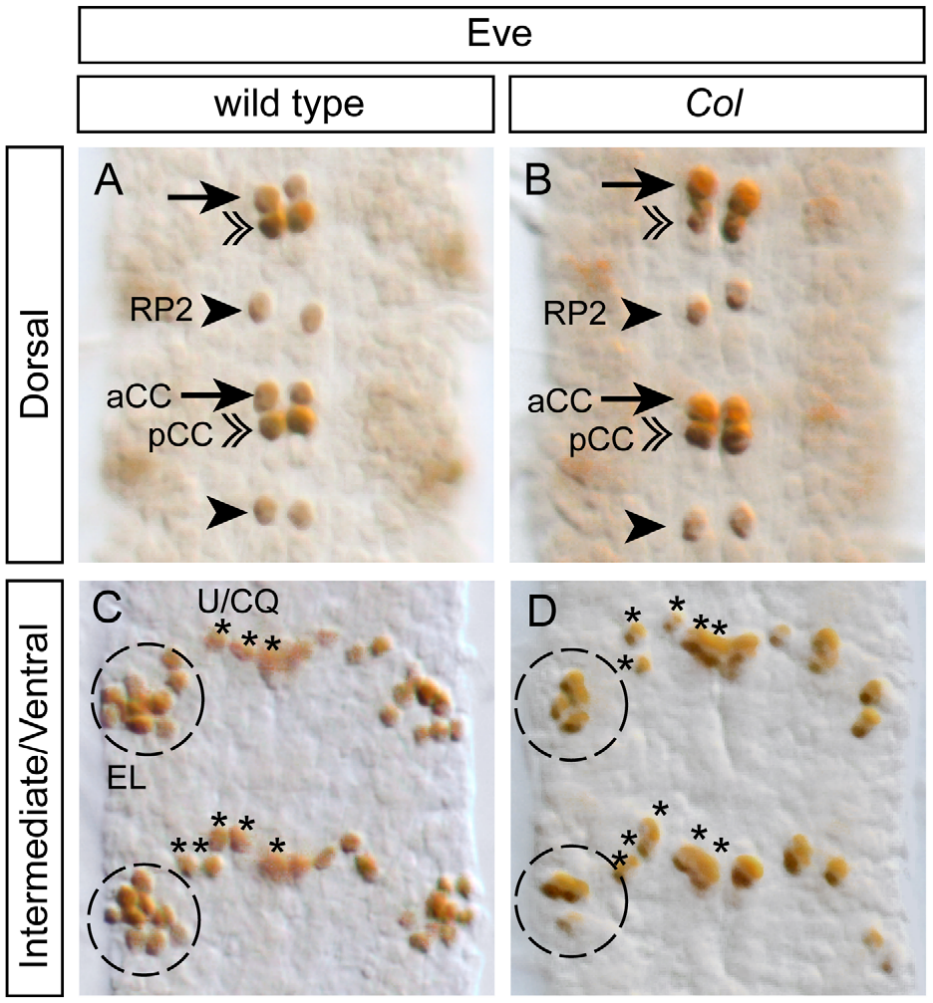
The differentiation of a subpopulation of EL interneurons is impaired in *Col* mutants. (A, C) In stage 15 wild type embryos, Eve staining allows for the identification of the aCC and RP2 motoneurons (arrows and arrowheads respectively) and for the pCC interneuron (double arrowheads) in the dorsal region of the VNC (A). In the intermediate/ventral region of the VNC (C), Eve staining decorates the 5 U/CQ motoneurons (U1–5) (asterisks) and the cluster of 8/10 EL (Eve Lateral) interneurons (dashed circle). (B, D) In stage 15 *Col* mutant embryos, while the aCC, RP2, pCC and U/CQ neurons develop normally (B), the EL interneuron cluster (D) is reduced to 4/5 neurons in most of the segment analyzed. In all panels two consecutive segments are shown.

Previous studies have shown that *col* is required for the proper differentiation of the dAp interneurons in the VNC [30], and a subset of the so-called multidendritic neurons in the PNS [27], [28], [29]. Here, we questioned whether a function for *col* in neuronal differentiation can be extended to other neurons. We focused on the well characterized EL cluster which contains, in the wild-type, 9–10 Eve^+^ neurons (100% of the hemisegments, n = 50; Figure 6) among which 4–5 express Col (Figure 3D–D″). We used two different *col* loss-of-function mutants, *col^1^* (null mutation) and *col^2^* (strong hypomorphic allele) [20], to assess the function of *col* in the differentiation of the EL neurons. With both alleles, we found a significant reduction of the number of EL neurons expressing Eve (Figure 6). In *col^1^* homozygous embryos, we found 4–5 Eve^+^ neurons in 58% of the hemisegments and 2–3 Eve^+^ neurons in 42% of the hemisegments (n = 73); in *col^2^* homozygous embryos, we found 4–5 Eve^+^ neurons in 89% of the hemisegments and 2–3 Eve^+^ neurons in 11% of the hemisegments (n = 48). We did not observe any effect on other Eve expressing neurons (which do not express Col in the wild-type), such as the aforementioned Eve^+^ motor neurons and the Eve^+^ pCC interneurons (Figure 6).

**Figure 7:**
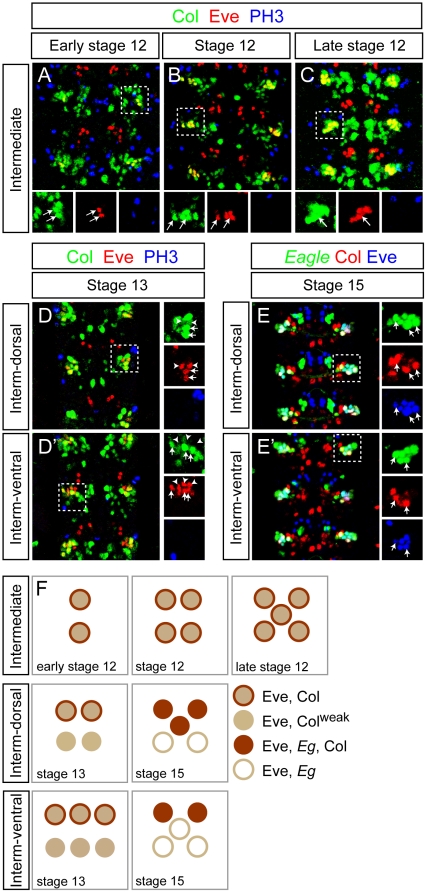
Temporal analysis of Col expression in the EL cluster. (A–C) In early stage 12 embryos (A) Col is expressed in the two first born Eve+ EL interneurons (arrows) per hemisegment. At stage 12 (B) and late stage 12 (C) Col expression overlaps with Eve respectively in 4 and 5 EL interneurons per hemisegment (arrows). Note that during these stages Col expression does not overlap with PH3 indicating that Col is only expressed in postmitotic cells in the intermediate region of the VNC. (D, D′) In the dorsal layer of the intermediate region (denoted Interm-dorsal) of stage 13 embryos two EL interneurons express high levels of Col (arrows) and two EL interneurons express weak levels of Col (arrowheads). In the ventral layer of the intermediate region (Interm-ventral) three EL interneurons express high levels of Col (arrows) and three EL interneurons express weak or undetectable levels of Col (arrowheads). As found for earlier stages Col expression does not overlap with PH3 in any cells. (E, E′) In stage 15 embryos most if not all EL interneurons express *eagle* (using *eagle-gal4*). Arrows indicate the EL interneurons that express Col. While three of the six EL interneurons express Col in the dorsal layer of the intermediate region two of the six EL interneurons express Col in the ventral layer of the intermediate region. In the close-up views presented here two Eve+ EL interneurons appear both in panels E and E′. (F) Diagram showing the time course expression of Col in the EL interneuron cluster from early stage 12 to stage 15. For simplicity note that in the schematic of the EL cluster at stage 15 five neurons are represented in each of the two layers that subdivide of the intermediate region of the VNC.

The number of missing Eve^+^ neurons in *col* mutants is superior to the number of cells expressing *col* in the wild-type in a significant fraction of embryos. In *col^1^*mutants, in 42% of the cases, there are 7 to 8 missing Eve*^+^* neurons while Col is expressed at most in 5 EL neurons in the wild-type. One obvious explanation for this paradoxical observation is that Col would be expressed in the neuroblasts or GMCs that produce the EL neurons and might therefore be involved in the generation of these neurons. To test this hypothesis, we performed a triple labelling with antibodies against Col, Eve, and PH3 on embryos from early stage 12 to stage 13 (when proliferating cells such as GMCs are present) to define whether some of the Col expressing cells in the EL cluster may be dividing cells. We did not find any co-localization between Col (or Eve) with PH3 indicating that Col (and Eve) are only expressed in post-mitotic neurons within this lineage (Figure 7A to 7D–D′), thus ruling out the possibility of a function of *col* in neuroblasts or GMCs. Interestingly we noticed that Col is expressed in all the early born EL neurons (up to five EL neurons in stage 12; Figure 7A to 7C). One possibility would be that the EL neurons expressing Col from stage 13 onwards (Figure 7D–D′, E–E′) would be the late born EL neurons and therefore that Col would be transiently expressed in most or all EL neurons. This view is further reinforced by the fact that we noticed faint Col expression in some Eve+ EL neurons in stage 13 embryos (these neurons are indicate by arrowheads in Figure 7D, D′ and express lower level of Col compare to the strong Eve+ EL neurons denoted by arrows; see Figure 7F for a summary of Col expression in the EL cluster).

We therefore conclude that *col* is required for the proper differentiation of the EL neurons. Combined with previously published studies [27], [28], [29], [30], our data indicate an involvement of *col* in the differentiation of various types of neurons both in the CNS and the PNS.

### An ortholog of *col* in the annelid *Platynereis, Pdu-coe,* is exclusively expressed in the developing nervous system

In order to determine whether the involvement of *col* in neural differentiation found in *Drosophila* can be extended to other protostomes, we cloned an ortholog of *col* in the polychaete annelid *Platynereis dumerilii*, by PCR using degenerated primers followed by RACE PCR with gene-specific primers. Sequence analysis and multiple alignments with known *coe* genes from representative metazoan species revealed that the cloned *Platynereis* gene encodes a protein displaying the three conserved domains found in all COE proteins, including the specific DBD domain (Figure S5). Protein sequence analyses show that the *Platynereis* ortholog clusters with the *coe* genes from other lophotrochozoans in the phylogenetic tree of the Coe family (Figure S5). We therefore named the cloned gene *Pdu-coe* (*Platynereis dumerilii* member of the Col/Olf1/EBF family of transcription factors). We isolated only a single *Coe* gene in *Platynereis*; however, as the genome of this annelid has not been fully sequenced yet, we cannot exclude that others may exist. Nevertheless, as members of the family have only been found as single genes in all non vertebrate species studied so far [3], [4], [5], [6], we expect that the one we cloned corresponds to the only *coe* gene present in *Platynereis*.

**Figure 8:**
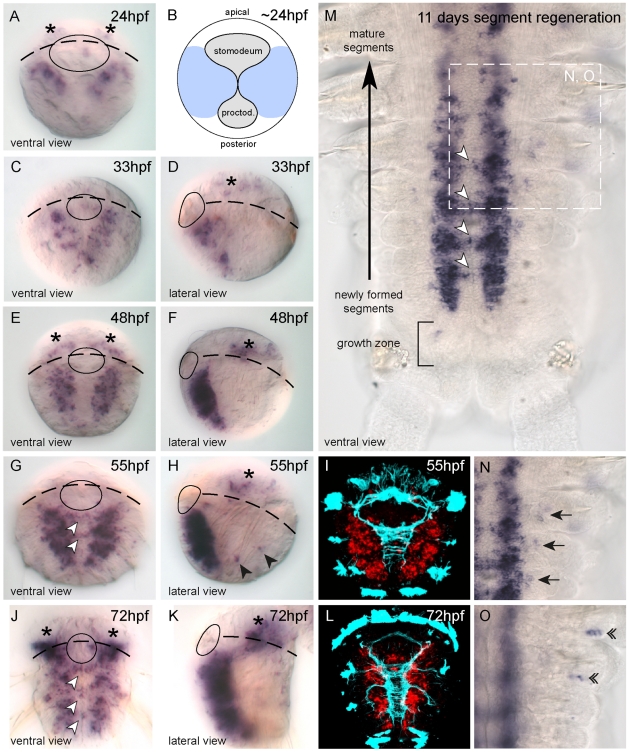
*Pdu-coe* is exclusively expressed in the developing nervous system in *Platynereis*. (A, C–K) Whole Mount *In Situ* Hybridization (WMISH) for *Pdu-coe* at 5 different embryonic stages. White arrowheads: commissural cells expressing *Pdu-coe*. Black arrowheads: Putative trunk PNS cells expressing *Pdu-coe* in the embryo. (B) Schematic depicting blastopore closure at 24 hpf, light blue indicates the ventral neurectoderm (based on SoxB expression). (I, L) *Pdu-coe* WMISH (red, confocal acquisition of a reflection signal, see methods) coupled with immunostaining against acetylated-tubulin (cyan). (M–O) Whole Mount *In Situ* Hybridization (WMISH) for *Pdu-coe* in a regenerating juvenile worm 11 days after cut. Anterior is up, posterior is down. (M) Whole regenerated segments, (N, O) focus on lateral cells expressing *Pdu-coe* on two different focal planes. Black arrows : *Pdu-coe* expressing cells probably belonging to the PNS in the ventral part of the appendages. Double arrowheads : *Pdu-coe* expressing cells probably belonging to the PNS in the dorsal part of the appendages. Stage and orientation are indicated on each panel.

We then analyzed the expression pattern of *Pdu-coe* during development by whole mount *in situ* hybridization (WMISH) at five developmental stages, ranging from 24hpf (hours post fertilization) to 72hpf, during which the VNC is formed. In 24hpf embryos, before closure of the blastopore, *Pdu-coe* expression is detected in two bilaterally-symmetrical areas in ventral neurectoderm which will give rise to the VNC (Figure 8A, B). From 33hpf to 72hpf, *Pdu-coe* expression is found only in the deep layers of the neurectoderm. The pattern extends posteriorly and laterally in correlation with extensive neurogenesis, marked by the expression of the neuronal markers *Pdu-elav* and *Pdu-syt* [34]. At 55hpf and 72hpf, transcripts are also detected in cells located across the ventral midline (Figure 8G, J; white arrowheads), in areas corresponding to the commissures of the VNC (Figure 8I, L). Moreover, a few scattered *Pdu-coe*-expressing cells are detected in the head area (Figure 8A, D, E, F, H, J, K; asterisks) belonging to the *Platynereis* brain [58]. Finally, *Pdu-coe* is also expressed in isolated lateral cells of the trunk (Figure 8H, black arrowheads) whose position suggests that they belong to the PNS. In contrast to what has been observed in other animals, we did not find any expression outside the nervous system and in particular in the developing mesoderm.

*Platynereis*, like most polychaete annelids, presents a biphasic development, with an early embryonic and larval development giving rise to a small 72hpf worm that bears only three chaetal segments (i.e. with appendages). The subsequent post-larval development involves the posterior addition of many chaetal segments (including their neural elements), from a subterminal growth zone containing stem cells, in a sequential manner throughout most of the life of the animal. This process is known as posterior growth and is mimicked by the capacity of juvenile worms to regenerate segments after a cut [59]. We thus analyzed *Pdu-coe* expression during segment regeneration by WMISH and found a pattern similar to that observed during larval development, i.e. expression in a large bilaterally symmetrical expression domain in the developing VNC and in commissural neurons (Figure 8M; white arrowheads). We also found a few *Pdu-coe*-expressing cells in the lateral ectoderm (Figure 8N; arrows and 8O double arrowheads), located in both the ventral and dorsal parts of the parapodes (the worm appendages) and probably belonging to the PNS. No expression in other tissues such as the mesoderm was observed. *Pdu-coe* expression is therefore, both during embryonic/larval and post-larval development, strongly associated with neurogenesis as transcripts are detected exclusively in developing neural tissues.

### *Pdu-coe* is expressed in diverse populations of neurons in the *Platynereis* VNC

We next tried to define more precisely the identity of the cells expressing *Pdu-coe* in the larval VNC. It has been shown that, consistently with what is observed in the vertebrate neural tube, the ventral neurectoderm of *Platynereis* is subdivided into several longitudinal progenitor domains defined by the partially overlapping expression of *pax* and *nk* genes, and from which originate conserved neuronal subtypes. From medial to lateral, we can thus define four progenitor domains characterized by the expression of *Pdu-nk2.2*+*Pdu-nk6*, *Pdu-nk6*+*Pdu-pax6*, *Pdu-pax6*+*Pdu-pax3/7*, and *Pdu-pax3/7* alone, respectively. Each domain will give rise to specific types of neurons: for example, the *Pdu-nk2.2*+*Pdu-nk6-*expressing progenitors will give rise to serotoninergic neurons while the *Pdu-nk6*+*Pdu-pax6-*expressing progenitors will produce *Pdu-hb9^+^* cholinergic motor neurons [33]. In addition to this medial-lateral axis, the existence of an apico-basal (superficial-internal) organization of the ventral neurectoderm has also been described [34], [35]. The most superficial (apical) layers of the ventral neurectoderm contain proliferative and undifferentiated progenitors expressing proneural genes such as *Pdu-achaete-scute* and *Pdu-neurogenin*, whereas intermediate ones are composed of post-mitotic cells undergoing differentiation and expressing early differentiation markers such as *Pdu-elav*. Finally, the deepest layers contain fully differentiated neurons expressing late differentiation markers such as *Pdu-syt*.

We used several of these markers to precisely characterize the expression pattern of *Pdu-coe* along both the medial/lateral and the apical/basal axes of the VNC neurectoderm. To avoid the many technical problems usually encountered with double fluorescent *in situ* hybridizations (such as the weakness and non-reliability of the fluorescent signals), we used a technique of pattern registration allowing *in silico* alignments of gene expression patterns, by taking advantage of the important reproducibility of *Platynereis* development and the highly stereotypic organization of the axonal scaffold of the larvae [58], [60], [61]. This method consists in independently acquiring images of the expression of different genes by whole-mount reflection confocal laser-scanning microscopy [62] on distinct larvae of the same batch/stage, reconstructing the patterns in 3D, and aligning them *in silico*, using the axon network of the VNC (revealed by immunostaining) as a reference (see Methods for further details). As validation of our method′s accuracy, we observed a good overlap between signals obtained for the same gene in different individuals and found a good agreement between double-labelling obtained by the *in silico* registration method and those obtained by double-fluorescent WMISH (Figure S6). While the expected variability of patterns and morphology between two distinct larvae prevents single cell resolution analysis; we can easily and accurately visualize complementarity and/or overlapping expression domains.

**Figure 9:**
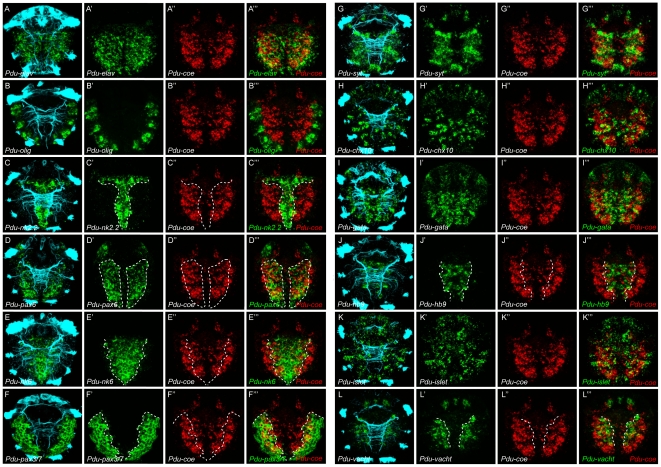
*Pdu-coe* is expressed in neurons which emerge from several different neural progenitor domains along the medial-lateral axis. Expression pattern registration for *Pdu-coe* (*red*) and *elav* (green, A–A‴), *olig* (green, B–B‴), *nk2.2* (green, C–C‴), *pax6* (green, D–D‴), *nk6* (green, E–E‴), *pax3/7* (green, F–F‴), *synaptotagmin* (*syt,* green, G–G‴), *chx10* (green, H–H‴), *gata123* (*gata,* green, I–I‴), *hb9* (green, J–J‴), *islet* (green, K–K‴), or *vacht* (green, L–L‴). Cyan: immunostaining against acetylated tubulin. All panels presented are ventral views of 55hpf embryos.

**Figure 10:**
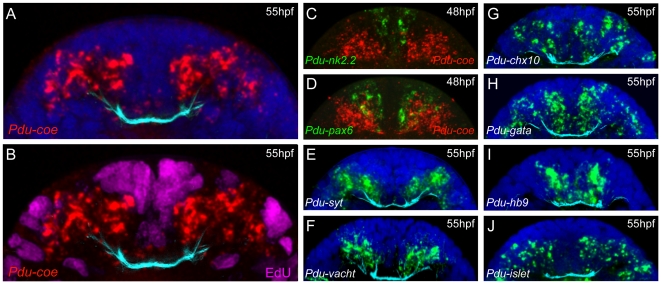
Expression of *Pdu-coe* is only found in post-mitotic neural cells and overlaps with that of several neuronal-identity genes. (A, B) WMISH for *Pdu-coe* (red) coupled with 30 min EdU incorporation (magenta on B) on the same 55hpf larvae. (C, D) Double fluorescent WMISH for *Pdu-coe* (*collier*, red) and *nk2.2* (C, green) or *pax6* (D, green). (E–J) WMISH for markers of various neuron populations (green). Cyan: immunostaining against acetylated tubulin. Blue : nuclear Hoechst staining. All panels presented are confocal transverse sections through the neurectoderm of 48hpf (C, D) or 55hpf (all others) embryos.

Our data first indicate that the ventral *Pdu-coe* expression domain is restricted to the developing VNC, as it completely overlaps with the *Pdu-elav* expression domain (Figure 9A–A‴), and directly abuts that of *Pdu-olig* (Figure 9B–B‴) which is expressed in the lateral ectoderm immediately adjacent to the VNC [35]. We then compared the expression domain of *Pdu-coe* with those of the *nk* and *pax* genes. It is important to note that *Pdu-coe* is expressed in the deep internal layers of the VNC (therefore probably in neurons) whereas the *pax* and *nk* genes are expressed mainly in the most superficial ones (therefore in progenitors), as revealed by transverse sections (Figure 10A,C,D). Overlaps of expression domains, as viewed in ventral views, will thus not reflect a real co-expression at the cellular level, but indicate from which progenitor domain(s) *Pdu-coe*-expressing cells are likely to be produced. We found that the expression domain of *Pdu-nk2.2* directly abuts that of *Pdu-coe* on its most medial border; the patterns are thus complementary except for the few *Pdu-coe* expressing cells localized along the axonal commissures (Figure 9C–C‴). Reciprocally, the *Pdu-coe* domain almost entirely overlaps with that of *Pdu-pax6* (itself complementary to *nk2.2*; [33]) and extends slightly more laterally (Figure 9D–D‴). The *Pdu-nk6* and *Pdu-pax3/7* expression domains partially overlap with *Pdu-coe*, in medial and lateral region, respectively (Figure 9E–E‴, F–F‴). *Pdu-coe*-expressing cells thus emerge from several different progenitor domains known to produce different types of neurons.

Given its expression in the deep layers of the neurectoderm, and according to previous observations about the organization of this tissue [33], [34], *Pdu-coe* seems to be expressed in the post-mitotic daughter cells of *pax*- and *nk*-expressing progenitors, and thus in various neuronal types. To assess this hypothesis, we first addressed whether *Pdu-coe*-expressing cells are indeed post-mitotic cells. We detected cells in S-phase using the EdU (5′-ethynyl-2′-deoxyuridine**)** proliferation assay on 55hpf larvae. This very sensitive method is much less harmful to the tissues than the most commonly used BrdU [63], thus allowing to perform both WMISH and antibody staining in addition to EdU labelling. Using this original technique, we directly compared the expression domain of *Pdu-coe* with the cell proliferation profile of the ventral neurectoderm and found no overlap (Figure 10B), indicating that *Pdu-coe* is only expressed in post-mitotic cells. We then compared the *Pdu-coe* expression pattern with that of several neuronal differentiation markers [33], and found extensive overlaps with the expression domains of all these genes, namely the general neuronal marker *Pdu-syt* (Figure 9G–G‴), the interneuron markers *Pdu-chx10* and *Pdu-gata123* (henceforth simplified as *Pdu-gata*) (Figure 9H–H‴,I–I‴), and the putative motor neuron markers *Pdu-hb9*, *Pdu-islet* and *Pdu-VAChT* (Figure 9J–J‴ to 9L–L‴). Contrary to *pax* and *nk* genes, these genes are expressed by differentiating neuronal cells located in the deep layers of the neurectoderm that co-express *Pdu-coe* (Figure 10A,E–J).

Taken together, our data indicate that *Pdu-coe* is exclusively expressed in the developing nervous system. In the VNC, its expression is only found in post-mitotic cells originating from several progenitor domains, and expressing various neuronal differentiation markers. *Pdu-coe* is therefore expressed, as its *Drosophila* ortholog *col*, in several subpopulations of neurons and may therefore be involved in their differentiation.

## Discussion

In this article, we report the fine characterization of *coe* gene expression in the CNS of two protostomes, the insect *Drosophila melanogaster* and the annelid *Platynereis dumerilii*. To our opinion, the use of these two species provides particularly pertinent data to envision *coe* gene evolution, as they belong to the two subgroups of protostomes (Ecdysozoa and Lophotrochozoa). Together with previously published vertebrate studies, our study thus allows the comparison of data from the three main bilaterian lineages. In addition *Drosophila* and *Platynereis* are both segmented animals that display different modes of development. Indeed, while in *Drosophila* the segments that constitute the whole CNS of the larvae are all formed in a single phase during embryogenesis, in *Platynereis* only three segments are first formed within a three days period. Afterwards the others segments are progressively added over a period of several months until the worm reaches its sexual maturity. Finally, the two species show differential modes of CNS patterning: whereas in *Platynereis* the CNS is subdivided into large domains from which emerge specific neural cell types (similarly to vertebrates) [33], [34], [35], *Drosophila* CNS patterning is mainly based on cell-to-cell specification processes in which every neuroblast (even neighbouring ones) express different combinations of developmental genes that control their identity [64].

### *col* is involved in the differentiation of interneurons in the *Drosophila* VNC

**Figure 11:**
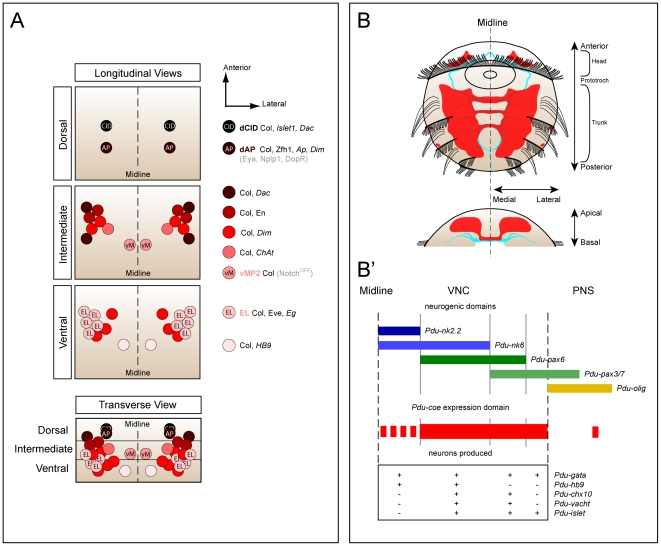
Summary of Coe expression data in the CNS of *Drosophila melanogaster* and *Platynereis dumerilii*. (A) Summary of Col expression in the VNC of *Drosophila* stage 15 embryos. The expression of Col is depicted in respect to the different molecular markers used in this study.- see text for more details. (B) Summary of *Pdu-coe* expression in the 55 hpf embryo of *Platynereis*. *Pdu-coe* expression domain is depicted in red, the developing VNC is represented in cyan. Top picture : ventral vew, bottom picture : transverse section. (B′) Schematic depicting the *Pdu-coe* expression domain in respect to the neurogenic domains determined by the combinatorial expression of *pax*/*nk* genes and the subsequently produced neurons defined by the molecular markers they express.

While *col*, the single *Drosophila* member of the *coe* family, has been isolated and shown to be expressed in many cells of the VNC 15 years ago [19], very little was known about the identity of the cells that express this gene and its function during CNS development, besides the fact that it is expressed in the thoracic dAp peptidergic interneurons and required for their proper differentiation [30]. Here we provide a detailed characterization of the expression pattern of Col in the VNC and show that it is mostly expressed in neurons, from early stage 12 until larval stages – at stage 14 we found Col to be expressed in about 50 neurons per hemisegment, i.e., about 10% of the total number of neurons. We observed that Col expression is excluded from glial cells and all known motor neurons, indicating that it is only expressed in interneurons. We used a large set of neuronal markers to define the identity of these neurons and, found Col to be expressed in diverse populations of interneurons, as seen by its co-expression with various neuronal-specification genes such as Eve, *eagle*, *dachshund*, Engrailed, *apterous*, *islet*, and Zfh1 (Figures 2, 3 and 11A).

We pointed out that some of the Col expressing cells correspond to previously characterized interneurons, such as the dAp neurons [30] in which Col is co-expressed with *apterous* and Zfh1, the EL neurons [47], [48] in which Col is co-expressed with Eve and *eagle*, and the vMP2 neuron [54] for which Col becomes a reliable marker. Besides, we also identified a previously uncharacterized Col expressing neuron, we named dCID, located close to the dAp neuron and characterized by the co-expression of Col, *islet*, and *dachshund*. The cell bodies of Col expressing neurons occupy diferent locations in the VNC and using *col-gal4* they are seen extending their axons to various locations within the CNS confirming both that they belong to highly different types of interneurons and that they are not motor neurons. We wondered whether these neurons may nevertheless share some common features, such as their neurotransmitter phenotype. We addressed this hypothesis using a large set of molecular markers (Figures 4 and 5), and found that 8–10 Col expressing neurons per hemisegment are cholinergic, as seen by the expression of *ChAT* [65] which encodes the choline acetyltransferase enzyme, and 6–7 are likely to be peptidergic, as seen by their expression of *dimmed*, a gene controlling peptidergic differentiation [53]. Although the neurotransmitters used by the remaining Col expressing neurons are still unknown, we can conclude that there is no evidence for a correlation between Col expression in a neuron and the acquisition of a specific neurotransmitter phenotype. Our data therefore indicate that Col is expressed in diverse subpopulations of interneurons that are highly heterogeneous. Indeed Col-expressing interneurons display differential properties such as: i) positions of their cell bodies in the VNC, ii) axonal projection patterns within the CNS, iii) molecular identities and iv) neurotransmitter phenotypes (Figure 11A). Whether these neurons may have some shared properties, beyond their expression of Col, remains to be defined.

This raises the question of the function of Col in the developing VNC. We focused on the EL interneurons which can be easily visualized by their expression of Eve [47]. Using *col* loss-of-function mutants, we found that *col* is required for the expression of Eve in these neurons (Figure 6), and thus probably necessary for their proper differentiation. This is reminiscent to what has been observed in the case of the dAp neurons [30] : in these neurons, *col* is required for the expression of several genes (*apterous*, *eye absent*, *dimmed*, *dopamine D1 receptor*, and *Neuropeptide like precursor protein 1*), which are themselves required for specific differentiation features including axonal pathfinding and the capacity to produce active neuropeptides. As a consequence, in *col* mutant embryos, dAp differentiation is impaired [30]. *col* function has also been described in a third type of neurons, a particular class (class IV) of multidendritic (md) sensory neurons, which display the most extensive and complex dendritic arborizations among md neurons [27], [28], [29]. There, *col* regulates the expression of at least two target genes, *pickpocket* and *spastin*, which are required to increase dendritic arbor outgrowth, a specific differentiation property of class IV as compared to other md neurons. Taken together, these data suggest that *col* controls the acquisition of specific features by acting on the expression of different sets of target genes in various populations of differentiating neurons. This capability to regulate the expression of different genes in different cells is probably due to the fact that, as suggested by previous studies [21], [30], Col may act mainly in combination with other transcription factors and therefore regulate its different targets in a context-dependent manner, depending on the presence/absence of its partners.

### *Pdu-coe* is expressed in differentiating neurons in the VNC of the annelid *Platynereis*

After having established the neuronal expression pattern of the *Drosophila col,* we aimed to delineate whether reminiscent expression patterns may be found in another segmented organism that exhibits a different mode of development. We thus identified and cloned a member of this family in the annelid *Platynereis dumerilii.* This gene, named *Pdu-coe* is expressed exclusively in developing neural territories of both embryos and juvenile worms. We combined WMISH and EdU labelling to show that *Pdu-coe* is exclusively expressed in post-mitotic cells in the developing VNC, and noticed that the *Pdu-coe* expression domain covers most of the post-mitotic VNC area (Figure 11B). To precisely characterize the expression pattern of *Pdu-coe* in the VNC, we have used a method of *in silico* alignment of gene expression patterns obtained by WMISH that allowed us to compare the expression of *Pdu-coe* with that of many genes previously shown to be involved in the development of the *Platynereis* VNC [33], [34], [35]. We found that *Pdu-coe* is expressed in many cells, probably neurons as they also express neuronal differentiation genes, such as *Pdu-elav* and *Pdu-syt*, which arise from different neural progenitor domains and express several different neuronal-specification genes. Indeed, it has been previously shown that the *Platynereis* neurectoderm can be subdivided into several domains characterized by the expression of specific *nk* and *pax* genes : from medial to lateral, there are 4 domains, *Pdu-nk2.2* + *Pdu-nk6*, *Pdu-nk6* + *Pdu-pax6*, *Pdu-pax6* + *Pdu-pax3/7*, and *Pdu-pax3/7* alone [33]. We found *Pdu-coe* expressed in neural cells emerging from the three most lateral domains, but excluded from medialmost ones (*Pdu-nk2.2* + *Pdu-nk6*), except for neurons located along the commissures (Figure 11 B′). However, our observations did not allow us to determine whether these neurons actually originate from the *Pdu-nk2.2* + *Pdu-nk6* domain or if they differentiate in more lateral domains and migrate toward the midline to achieve their final position.

Each of these four domains has been shown to produce specific neuronal types characterized by the expression of neuronal specification genes, such as *Pdu-chx10*, *Pdu-hb9*, *Pdu-islet* [33], and *Pdu-gata*. We found that the *Pdu-coe* expression domain overlaps with those of all these genes (Figure 10). Although our method of *in silico* pattern registration does not allow to demonstrate whether the studied genes are actually expressed in the same cells, it is much likely that *Pdu-coe* is co-expressed with each of these genes in, at least, some of the VNC cells. Our observations therefore suggest that *Pdu-coe* is expressed in most neurons of the VNC (some of the most medial cells expressing *Pdu-gata* and possibly *Pdu-hb9* are not included in the *Pdu-coe* domain), and that these neurons constitute diverse subpopulations characterized by the expression of specific neuronal specification genes (Figure 11B′). Given our observation that in *Drosophila* Col expression is excluded from motor neurons, an important issue is nevertheless to know whether *Pdu-coe* is expressed in *Platynereis* motor neurons. Unfortunately, we are unable to give a definitive answer to this question. Based on comparisons with vertebrates, it has been suggested that *Pdu-hb9^+^* cholinergic neurons emerging from the *Pdu-nk6* + *Pdu-pax6* domain are the *Platynereis* motor neurons [33]. We found that some *Pdu-coe* expressing cells derive from the *Pdu-nk6* + *Pdu-pax6* domain and likely co-express *Pdu-hb9* and *Pdu-VAChT* (a marker of cholinergic neurons), suggesting that *Pdu-coe* may be expressed in motor neurons. However, we have to consider that all cholinergic neurons are not necessarily motor neurons (some of them may be interneurons as it is the case in *Drosophila*), that all motor neurons are not necessarily cholinergic (motor neurons are glutamatergic in *Drosophila* for example), and that *hb9* is expressed in motor neurons and interneurons, both in *Drosophila* [41], [66] and in vertebrates [67], [68]. Further studies would therefore be required to define the exact position and number of motor neurons in *Platynereis* and to determine whether these neurons express *Pdu-coe*.

### Comparative analysis of *coe* genes in animals: conservation of ancestral functions versus multiple recruitments

One striking property of the *coe* gene family is its strong conservation in animals: *coe* genes have been found in all animal species in which these genes have been searched, usually as single gene, and encode proteins with a very high sequence similarity [3], [5], [6]. It is therefore tempting to hypothesize that these genes also share well conserved functions. One such conserved function might be during neurogenesis as all *coe* genes studied to date are expressed in the developing nervous system. However, neurogenesis is a complex process that involves many steps and many genes - to define whether *coe* genes may play homologous roles during nervous system development in various animal lineages requires defining their function more precisely during the neurogenesis of different animals. Our work combined with some previously published data sheds light on a striking correlation between *coe* genes and one particular aspect of the neurogenesis process, namely the neuronal differentiation. As discussed above, *Drosophila col* is likely involved in the differentiation of various types of interneurons. In *Platynereis*, *Pdu-coe* is expressed in most neuron classes of the VNC and might participate in their differentiation. In another annelid, *Capitella teleta*, *coe* is also broadly expressed in the developing VNC [6] in a pattern reminiscent to the one found in *Platynereis*. *unc-3*, the *C. elegans coe* gene, is required for some aspects of the differentiation of both CNS and PNS neurons [31], [32]. In vertebrates, *Ebf* genes, the vertebrate *coe* orthologs, are expressed in most post mitotic differentiating neurons of the CNS and required for at least some aspects of their differentiation [10], [11], [12], [13], [14], [15], [16], [17]. Closely related to the vertebrate lineage, the ascidian *Ciona intestinalis* (urochordate) shows expression of *coe* in a few cells of the neural plate [69] and all differentiating neural precursors of the visceral ganglion (a structure proposed to be homologous to the vertebrate spinal cord) [70]. A recent study also suggests that the *coe* gene in the sea urchin (echinoderm) *Strongylocentrotus purpuratus* is involved in the differentiation of some neurons belonging to the apical ganglion of the larva [6]. Our data therefore reinforce the hypothesis that an involvement of *coe* genes in neuronal differentiation is ancestral to bilaterians. As *coe* genes are likely to be also expressed in differentiating neuronal cells in non bilaterians, such as a cnidarian and a ctenophore [4], [6], a role in neuronal differentiation is probably an ancestral feature of the *coe* transcription factors family, which may have arisen early in metazoan evolution. *coe* genes have also been identified in the sponge *Amphimedon queenslandica* and the placozoan *Trichoplax adhaerens* [5], [6] which lack true neurons - it would be interesting to define the expression pattern of *coe* genes in these two species.

Another possible conserved function of *coe* genes may be during mesoderm development [6]. *Drosophila col* is known to be expressed in a few muscles and required for their subtype specification [21], as well as in the lymph gland where its function is required for the maintenance of hematopoietic progenitors, a prerequisite to the formation of lamellocytes (immune cells involved in response to parasites) [25]. In mice, *ebf* genes are key factors controlling B-cell lymphopoiesis [71], [72], however, while mouse *ebf* genes have a cell-autonomous function in B-cell development, *Drosophila col* is not expressed in the lamellocytes and controls their differentiation in a non–cell-autonomous manner [25]. In addition, the determination of homologies between vertebrate and non vertebrate blood cells is still challenging [73]. Nevertheless, vertebrate *ebf* genes can also act in a non cell-autonomous manner as shown during mouse bone development, where *ebf2* is expressed in osteoblast progenitors and acts as a regulator of osteoblast-dependent osteoclast differentiation [74]. Another important mesoderm related function of *coe* genes has been recently uncovered in the urochordate *Ciona intestinalis*. Here, *Ci-coe* acts as a critical determinant in the specification of atrial siphon muscles, a cell lineage that arises from the same progenitors as cardiomyocytes [75]. Furthermore, expression of the *Xebf2* gene in the anterior lateral mesoderm of *Xenopus tropicalis* embryos suggests that *coe* genes may be a conserved determinant of cardiopharyngeal mesoderm development in chordates [75]. However, mesodermal expressions were not found in another chordate, the Amphioxus [18] or in the non-chordate deuterostome, the sea urchin *S. purpuratus* [6]. Regarding protostome species, a recent study reports the expression of *coe* genes in mesodermal derivatives in two annelids and a mollusc [6]. While in the annelid *Capitella teleta*, this expression seems to be localized in the mesodermal stem cells of the trunk, in the other annelid *Chaetopterus* and in the mollusc *Haliotis asinina* the identities of the mesodermal *coe*-expressing cells are unknown. In this study expression of *Pdu-coe* in the mesoderm was not detected in *Platynereis*. It is therefore impossible so far to define whether the existence of some mesodermal expression of coe *genes* is ancestral to lophotrochozoans (and in this case, this expression would have been lost in *Platynereis* or would be endowed by another uncharacterized *coe* gene) or whether mesodermal expressions have been independently acquired in some lophotrochozoan lineages. Further studies will therefore be required to test the hypothesis of ancestral function of *coe* genes in the development of mesodermal derivatives and particularly the stimulating hypothesis of *coe* genes being involved in the differentiation of some ancestral bilaterian immune cells [25].

More generally, members of the *coe* family are involved in the differentiation of an astonishing diversity of cell types among bilaterians and even within one species. In *Drosophila*, for example, in addition to CNS and PNS cells, muscles, blood cells, *col* is also involved in the patterning of the wing disc downstream of the Hedgehog signalling pathway [23], [24]. Such involvement in appendage patterning and/or Hedgehog pathway is not found in other species. We therefore propose that several recruitments occurred independently during bilaterian evolution, reflecting a high plasticity of Coe proteins related to their combinatorial mode of action. We hypothesize that Coe proteins are transcription factors that have been co-opted several times in pre-existing regulatory networks to ensure robust gene expression regulation. In this view, we would expect to find species – or lineage-specific expression sites of *coe* genes when looking at additional species – the expression in the foregut and mesodermal stems cells of *Capitella teleta* [6], not found in the other annelid *Platynereis,* may be such a specific expression. At the molecular level, our hypothesis would imply that target genes of Coe proteins may vary largely in different cells and in different species and that the specificity in Coe target gene recognition would depend on the interaction with other transcription factors or other protein cofactors. While some data in agreement with this proposal have been found in *Drosophila* [21], [30], further test of this hypothesis awaits more systematic searches for Coe target genes and Coe interactors.

## Materials and Methods

### Fly stocks, breeding culture, and embryo collection

*Drosophila melanogaster*: The stocks used as controls were either *y^-^w^-^* or *w^1118^* (Bloomington Stock Center). The *Barh1^LacZ^* enhancer trap line [76] is a specific marker for SNa motoneurons and dopaminergic neurons as previously reported in [45]. The Gal4 drivers used in this study are: *HB9^Gal4^* [66] (provided by J. B. Skeath and H. T. Broihier); *Ap^Gal4^* [77]; *Dac^Gal4^* [78]; *Eg^Gal4^* [52]; *dMP2^Gal4^* [39]; *Col^Gal4^* [26] (from M. Crozatier and A. Vincent); *Dim^Gal4^* [79]; *Chat^Gal4^* [65]; *TH^Gal4^* [51], *OK371^Gal4^* (denoted v*Glut^Gal4^* in the text) [80]. The *BarH1^Gal4^* driver [81] was previously described as a specific marker for in SNa motoneurons [45]. The reporter lines used are *UAS-mEGFP^F^* [82]; *UAS-nls-myc-EGFP* [83]; *UAS-CD8-GFP* [84]. Other stocks were *Islet-tau-myc-EGFP* [44]; U*AS-Notch^ICD^* [85] and *col^1^*, *col^2^* [29]. *Col* mutants were kept over a *CyO, Act-GFP* balancer chromosome. All genetic crosses were performed at 25°C. In genetic crosses where Gal4 drivers were used over night embryos collections were transferred to 29^o^C for at least 5 hours before dissection to insure robust expression.

*Platynereis dumerilii*: worms were obtained from a breeding culture established in the Institut Jacques Monod (Paris), according to the protocol of Dorresteijn et al [86]. Larvae and regenerating worms were fixed in 4% PFA in PBS + 0.1%Tween20 and stored at −20°C in methanol.

### Isolation of *Pdu-coe* and phylogenetic analyses

PCR using degenerated primers (described in [12]) on cDNAs from mixed larval stages was used to clone a 560 bp fragment of *Pdu-coe*. Larger fragments were subsequently amplified using SMART™ RACE cDNA amplification procedures with gene-specific primers. PCR products were TA cloned into the PCR2.1 vector (Invitrogen), and sequenced. The largest *Pdu-coe* clone (916 pb), corresponding to a partial cDNA, was then used as templates to produce RNA antisense probes for WMISH. Despite extensive efforts, we were unable to clone a full-length *Pdu-coe* cDNA. Accession number for *Pdu-coe* is GU169416. Multiple sequence alignments were built with ClustalW (Figure S7), using a large set of COE orthologs identified by BLAST (mainly from animal whose genome has been fully sequenced). Neighbour-joining (NJ) reconstructions were performed with the PAUP 4.0 program using the BioNJ algorithm and 1000 bootstrap replicates [87], [88]. Maximum likelihood (ML) analyses were performed with PhyML using the WAG model of amino acid substitutions [89] and 150 bootstrap replicates. Bayesian interference was performed using the Markov chain Monte Carlo method as implemented in MrBayes, using the WAG model. Two independent Markov chains were run, each including 3,000,000 generations Monte Carlo steps. The presented tree is a consensus of those obtained with methods mentioned above. Marginal probabilities at each internal branch were taken as a measure of statistical support.

### Whole-mount in situ hybridization (WMISH), Immunodetection, EdU cell proliferation assay

#### Drosophila melanogaster

Immunofluorescent and immunohistochemical stainings of dissected embryos and larval VNC were performed as described by Thor [90] using the following antibodies: mouse monoclonal antibody (mAb) anti-Col (1∶150; provided by M. Crozatier and A. Vincent), guinea pig anti-Col (1∶400; provided by S. Thor), anti-Elav (7E8A10) (1∶50), anti-Repo (8D12) (1∶80) anti-Engrailed/invected (4D9) (1∶50), anti-Eve (3C10) (1/40) (all from Developmental Studies Hybridoma Bank), rabbit anti-pMad [91] (1∶500; gift from P. ten Dijke), rabbit anti-Zfh1 [92] (1∶5000; gift from R. Lehmann), guinea pig anti-Dbx [50] (1∶1000; gift from JB. Skeath), rabbit anti-GAD1 [93] (1∶400; anti-DGAD1 antisera 818, gift from K. Broadie), rabbit anti-GFP (1∶4000; A-6455, Molecular Probes, Eugene), rabbit anti-beta-gal (1∶4,000; ICN-Cappel, Aurora, Ohio, United States) and rabbit anti-phospho-histone H3-Ser28 (PH3) (1∶4000; 07–145, Upstate/Millepore, Billerica, Massachusetts, United States). Prior to use, all primary polyclonal antibodies were pre-absorbed against early-stage wild-type embryos. Fluorescent secondary antibodies were obtained from Molecular Probes (Alexa 488, Alexa 594 used at 1∶500) or Jackson Labs (Cy3; 1∶2000) and (Cy5; 1∶1000). Immunohistochemical stainings were carried out using the Vectastain ABC Elite kit following manufacturers' protocol (Vector Labs).

#### Platynereis dumerilii

NBT/BCIP WMISH and double fluorescent WMISH were done as described in [93]. Embryos were counterstained with Hoechst and anti-acetylated tubulin antibody (Sigma T6793) at 1∶500. After NBT/BCIP staining, embryos were incubated with fluorescent secondary anti-mouse IgG Alexa Fluor® 488 conjugate (Invitrogen) at 1∶500. EdU proliferation assay was performed using the Clickt-iT® EdU kit (Molecular Probes), incorporation of EdU (200µM) was done for 30minutes directly in sea water.

### Microscopy, registration and images processing

#### Drosophila melanogaster

For all fluorescent immunostaining a Zeiss LSM 5 Confocal microscope (Zeiss, Oberkochen, Germany) was used to collect data. Confocal stacks were manipulated using the LSM 5 software and Adobe Photoshop (Adobe Systems, San Jose, California, United States). Double-labelled images were false colored using Photoshop for the benefit of color-blind readers.

#### Platynereis dumerilii

Bright field images were taken on a Leica microscope using DIC optics. Adjustment of brightness/contrast and Z projections were performed using the ImageJ software. Confocal images were taken with a Leica SP5 confocal microscope with a 63x oil immersion objective. For 3D pattern registration, confocal pictures were independently acquired on two distinct larvae of the same litter and stage. Signals acquired included whole mount reflection [62] and acetylated tubulin antibody staining marking the VNC network. Both signals were 3D reconstructed using the Amira software. Embryos were then aligned using the registration tool included in Amira, using only the reproducible VNC network antibody staining as a reference. Images presented in figure 8 are snapshots of the 3D reconstructed and aligned embryos, with only the reflection signals displayed (see Figure S6 for Registration tests).

## Supporting Information

Figure S1 ***Col*** **is expressed from early stage 12 to larval stages.** (A, A′) In early stage 12, Col expression overlaps extensively with expression of *Col^Gal4^* notably in the intermediate region of the VNC (A′). Note that in the dorsal and lateral region of the VNC (A) some Col expressing cells do not express *Col^Gal4^* suggesting that in these cells *Col* expression may be very transient. (B, B′) In stage L2, *Col^Gal4^* reveals that *Col* expressing cells are neurons (Elav+). Two, three or four consecutive segments are respectively shown in (A), (B) and (B, B′).
(TIF)

Figure S2 **Col expression in respect to motor neurons markers.** Stage 15 embryos stained with Col and different motor neuron markers. (A–B″) No overlap between Col and Zfh1 is found in any of the different regions of the VNC examined. (C–D″) Col and *HB9^Gal4^* expression only overlaps in one or two cells per hemisegment. These cells, located in the ventral region of the VNC, are pMad negative and thus are most probably interneurons. (E–F″) Expression of Col and *BarH1^Gal4^*, a specific marker for SNa (Segmental Nerve a) motor neurons is mutually exclusive.
(TIF)

Figure S3 **Col expression in respect to interneurons markers.** Stage 15 embryos stained with Col and Engrailed (A–B″) or Col and Dbx (C–E″). Col expression does not overlap neither with En nor with Dbx in the different regions of the VNC shown here.
(TIF)

Figure S4 **Col is expressed in the vMP2 interneurons and its expression can be induced in the dMP2 neurons using an activated form of Notch.** (A) In stage 15 wild type embryos, the *dMP2^Gal4^* line allows for the identification of the dMP2 neurons (arrowhead). Col is not expressed in dMP2 but in the dMP2 sibling neuron, vMP2 (double arrowhead) that is found in close association with dMP2 in a slightly more anterior and ventral location. (B) Using the *dMP2^Gal4^* line in association with *UAS-Notch^ICD^* expression of Col is induced in dMP2 while maintained in vMP2. (C) The MP2 neuroblast divides ones and gives rise to the sibling vMP2 (Notch^ON^) and dMP2 (Notch^OFF^) neurons.
(TIF)

Figure S5 **Phylogenetic relationship and structure of COE proteins from various metazoan species.** Phylogenetic tree of the COE protein family among Metazoans (consensus between Bayesian-Interference, Neighbour-Joining and Maximum-Likelihood analyses, see methods). Nodes marked by a dot are conserved in all three methods, colours and numbers indicate statistical support. From top to bottom at each node: Posterior probability for BI, Bootstrap value for NJ and ML. A black dot indicates supporting values all comprised between 90 and 100. The node corresponding to Urbilateria, the last common ancestor of Bilaterians, is indicated by a red circle.
(TIF)

Figure S6 **Controls for** ***in silico*** **alignment of expression patterns.** (A–D) *In silico* pattern registration for *Pdu-coe* WMISH performed on two distinct 55hpf embryos. (D) 3D reconstruction and registration of axonal scaffolds marked by immunostaining against acetylated tubulin in the two distinct aligned embryos. (E–H) Comparison between *in silico* pattern registration (E–G) and double fluorescent WMISH (H) for *Pdu-coe* and *nk2.2*. (I–L) Comparison between *in silico* pattern registration (I–K) and double fluorescent WMISH (L) for *Pdu-coe* and *pax6*. Cyan : immunostaining against acetylated tubulin. All panel are ventral views of 55hpf embryos, except H and L (48hpf).
(TIF)

Figure S7 **Multiple alignment used for phylogenetic analysis of COE proteins.** Pladum : *platynereis dumerilii* ; Capspl : *Capitella sp* ; Lotgig : *Lottia gigantea* ; Apimel : *Apis melifera* ; Nasvit : *Nasonia vitripennis* ; Dromel : *Drosophila melanogaster* ; Anegam : *Anopheles gambiae* ; Pedhumcor : *pediculus humanus corporis* ; Tricas : *Tribolium castaneum* ; Dappul : *Daphnia pulex* ; Caebri : *Caenorhabditis briggsae* ; Caeele : *Caenorhabditis elegans* ; Sackow : *Saccoglossus kowalevskii* ; Strpur : *Strongylocentrotus purpuratus* ; Braflo : *Branchiostoma floridae* ; Homsap : *Homo sapiens* ; Musmus : *Mus musculus* ; Galgal : *Gallus gallus* ; Brare : *Brachidanio rerio* ; Nemvec : *Nematostella vectensis* ; Triadh : *Trichoplax adherens* ; Ampque : *Amphimedon queenslandica*.
(TIF)
