# Supplementary figures and images for: Coe Genes Are Expressed in Differentiating Neurons in the Central Nervous System of Protostomes
Source: PLoS One. 2011 Jun 17;6(6):e21213. doi: 10.1371/journal.pone.0021213 (PMC3117877; doi:10.1371/journal.pone.0021213)

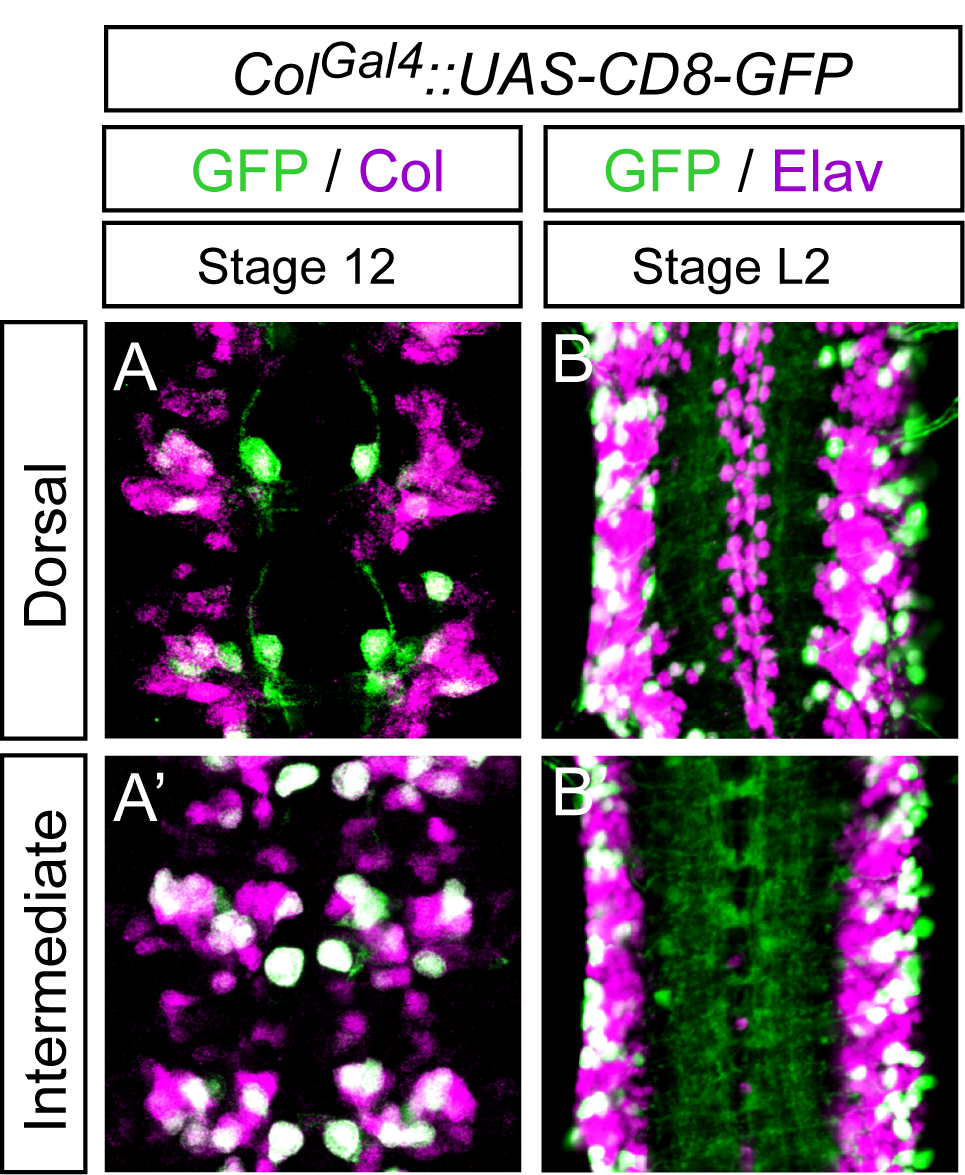

Supplement: Figure S1 — Col is expressed from early stage 12 to larval stages. (A, A′) In early stage 12, Col expression overlaps extensively with expression of ColGal4 notably in the intermediate region of the VNC (A′). Note that in the dorsal and lateral region of the VNC (A) some Col expressing cells do not express ColGal4 suggesting that in these cells Col expression may be very transient. (B, B′) In stage L2, ColGal4 reveals that Col expressing cells are neurons (Elav+). Two, three or four consecutive segments are respectively shown in (A), (B) and (B, B′). (TIF) [file pone.0021213.s001.tif]

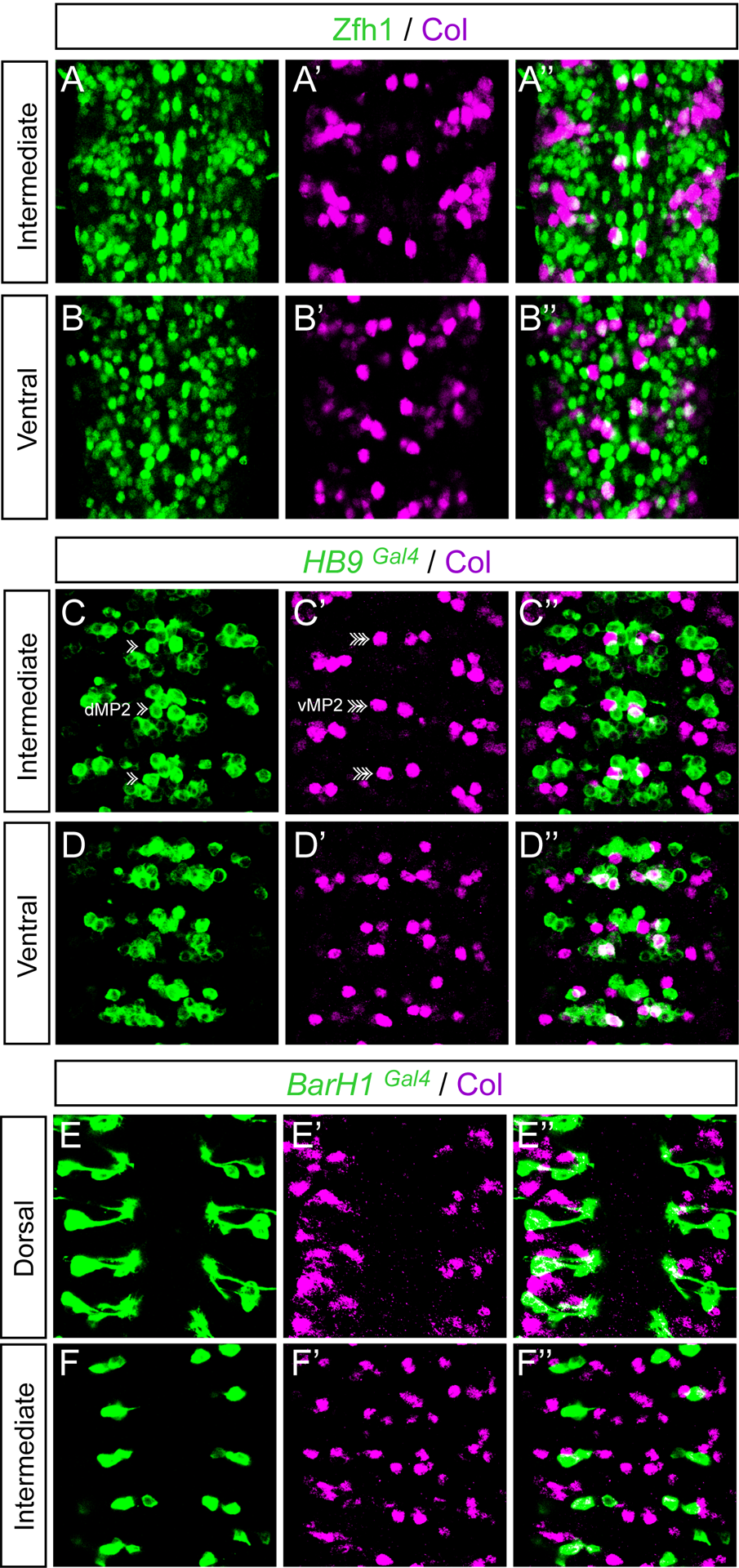

Supplement: Figure S2 — Col expression in respect to motor neurons markers. Stage 15 embryos stained with Col and different motor neuron markers. (A–B″) No overlap between Col and Zfh1 is found in any of the different regions of the VNC examined. (C–D″) Col and HB9Gal4 expression only overlaps in one or two cells per hemisegment. These cells, located in the ventral region of the VNC, are pMad negative and thus are most probably interneurons. (E–F″) Expression of Col and BarH1Gal4, a specific marker for SNa (Segmental Nerve a) motor neurons is mutually exclusive. (TIF) [file pone.0021213.s002.tif]

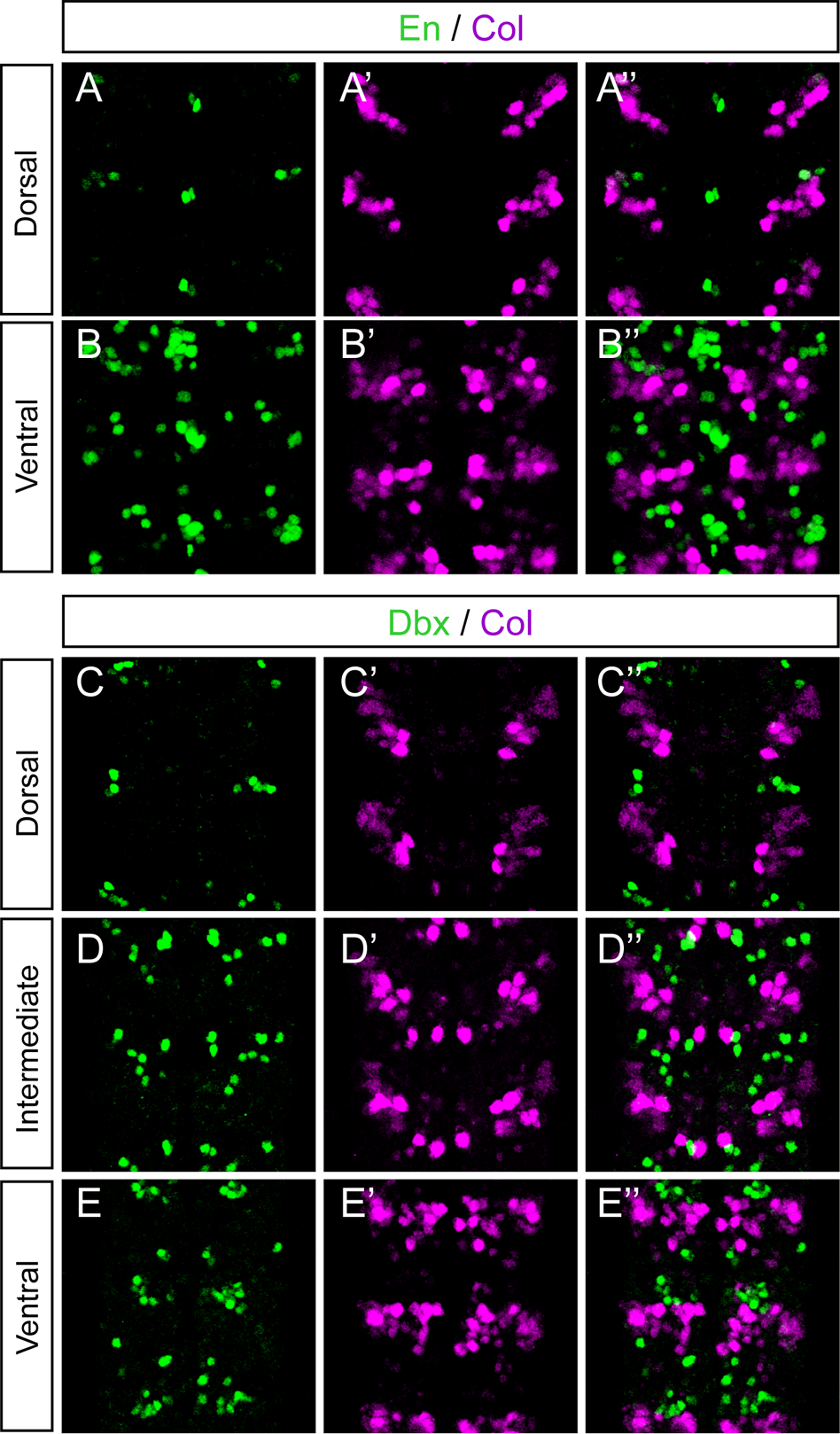

Supplement: Figure S3 — Col expression in respect to interneurons markers. Stage 15 embryos stained with Col and Engrailed (A–B″) or Col and Dbx (C–E″). Col expression does not overlap neither with En nor with Dbx in the different regions of the VNC shown here. (TIF) [file pone.0021213.s003.tif]

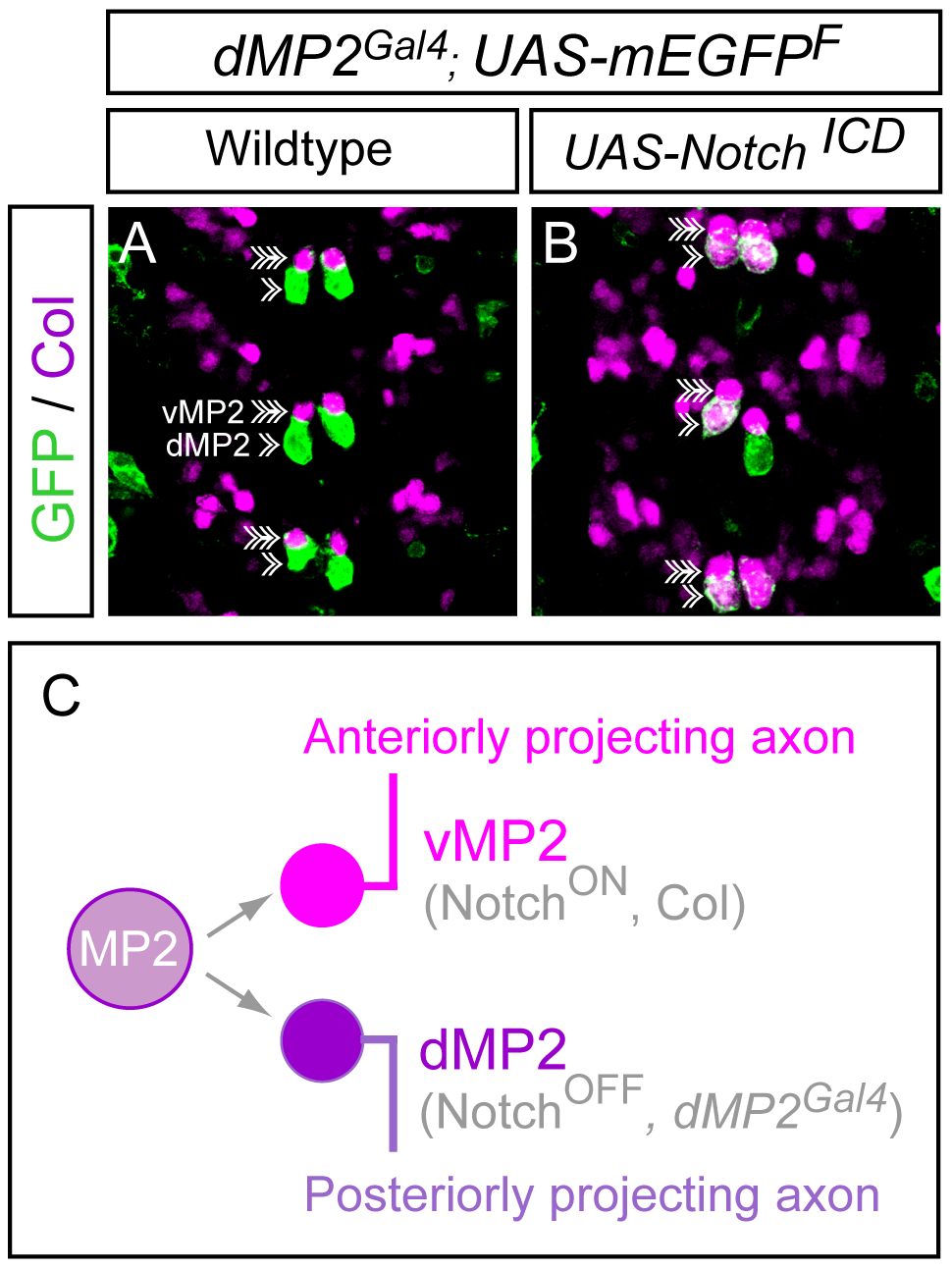

Supplement: Figure S4 — Col is expressed in the vMP2 interneurons and its expression can be induced in the dMP2 neurons using an activated form of Notch. (A) In stage 15 wild type embryos, the dMP2Gal4 line allows for the identification of the dMP2 neurons (arrowhead). Col is not expressed in dMP2 but in the dMP2 sibling neuron, vMP2 (double arrowhead) that is found in close association with dMP2 in a slightly more anterior and ventral location. (B) Using the dMP2Gal4 line in association with UAS-NotchICD expression of Col is induced in dMP2 while maintained in vMP2. (C) The MP2 neuroblast divides ones and gives rise to the sibling vMP2 (NotchON) and dMP2 (NotchOFF) neurons. (TIF) [file pone.0021213.s004.tif]

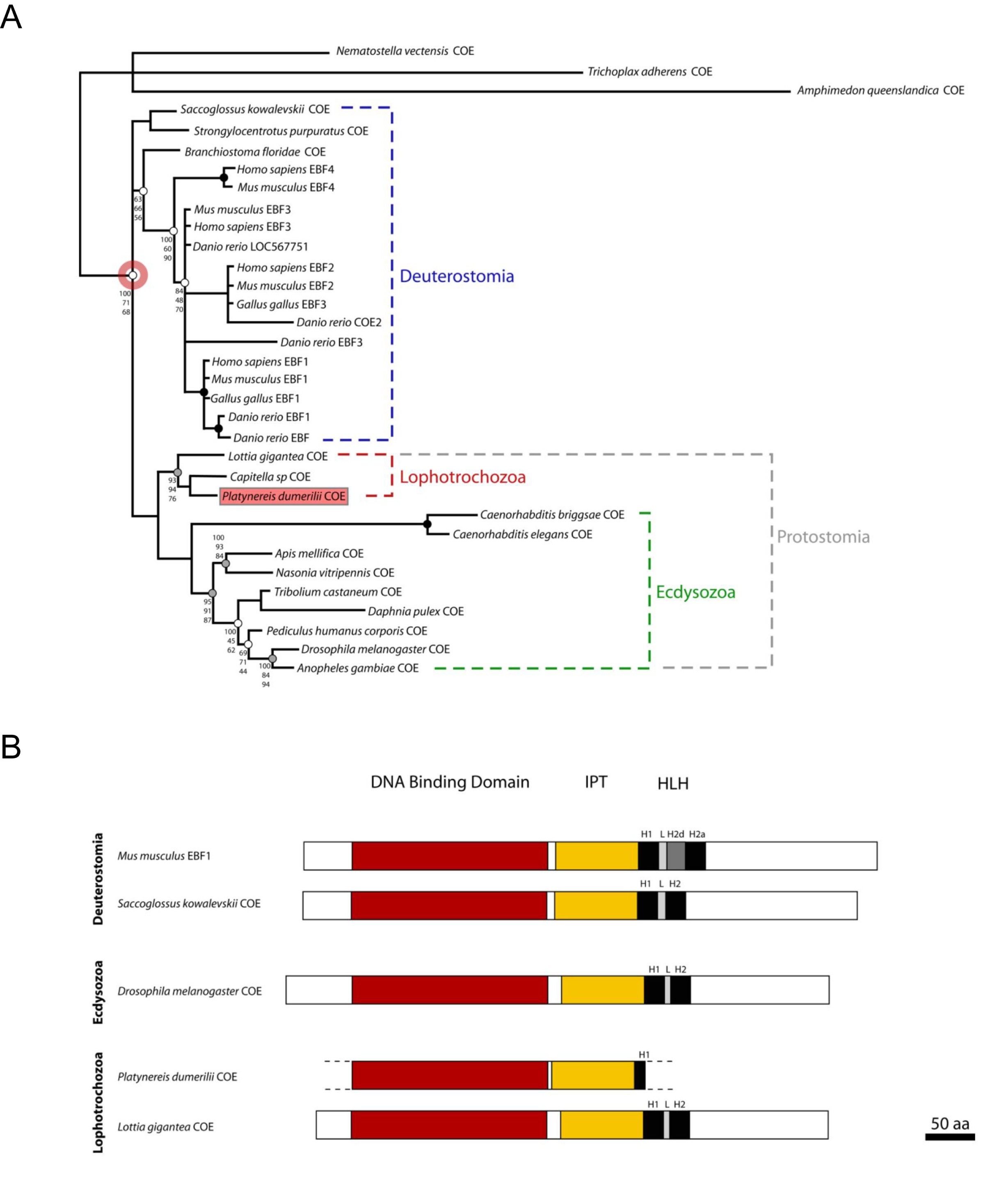

Supplement: Figure S5 — Phylogenetic relationship and structure of COE proteins from various metazoan species. Phylogenetic tree of the COE protein family among Metazoans (consensus between Bayesian-Interference, Neighbour-Joining and Maximum-Likelihood analyses, see methods). Nodes marked by a dot are conserved in all three methods, colours and numbers indicate statistical support. From top to bottom at each node: Posterior probability for BI, Bootstrap value for NJ and ML. A black dot indicates supporting values all comprised between 90 and 100. The node corresponding to Urbilateria, the last common ancestor of Bilaterians, is indicated by a red circle. (TIF) [file pone.0021213.s005.tif]

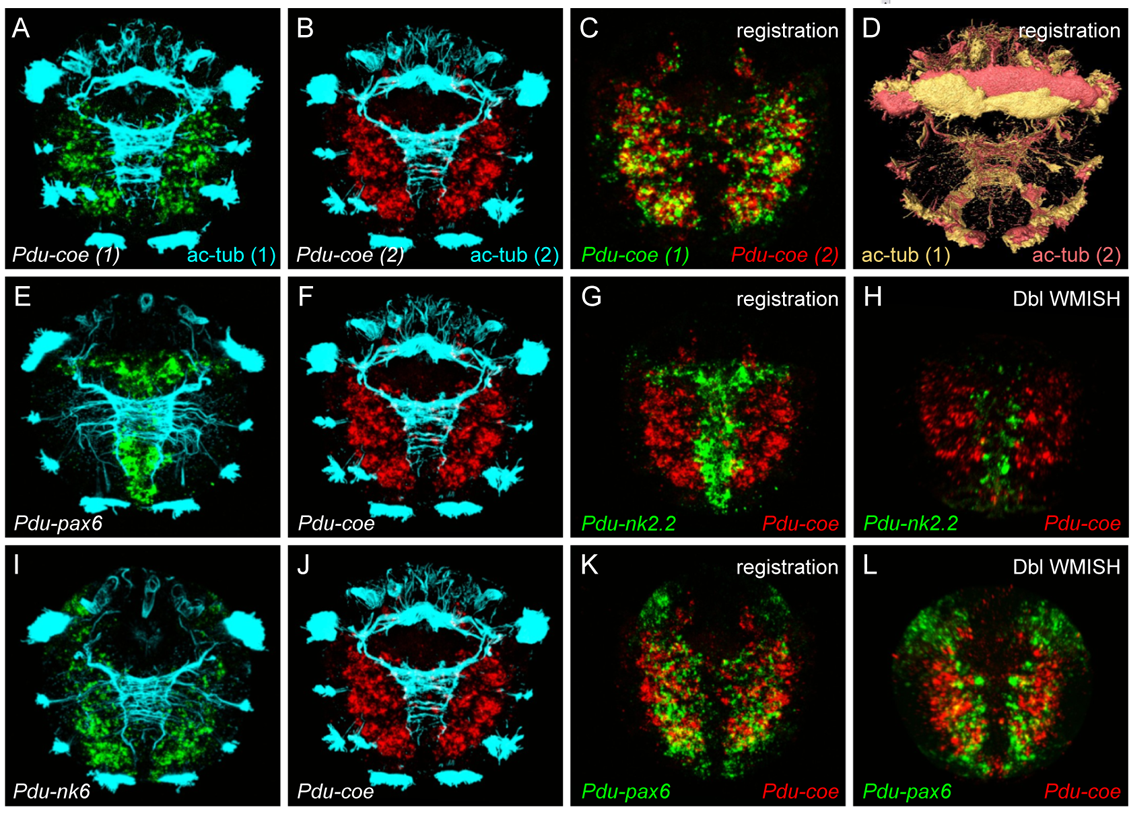

Supplement: Figure S6 — Controls for in silico alignment of expression patterns. (A–D) In silico pattern registration for Pdu-coe WMISH performed on two distinct 55hpf embryos. (D) 3D reconstruction and registration of axonal scaffolds marked by immunostaining against acetylated tubulin in the two distinct aligned embryos. (E–H) Comparison between in silico pattern registration (E–G) and double fluorescent WMISH (H) for Pdu-coe and nk2.2. (I–L) Comparison between in silico pattern registration (I–K) and double fluorescent WMISH (L) for Pdu-coe and pax6. Cyan : immunostaining against acetylated tubulin. All panel are ventral views of 55hpf embryos, except H and L (48hpf). (TIF) [file pone.0021213.s006.tif]

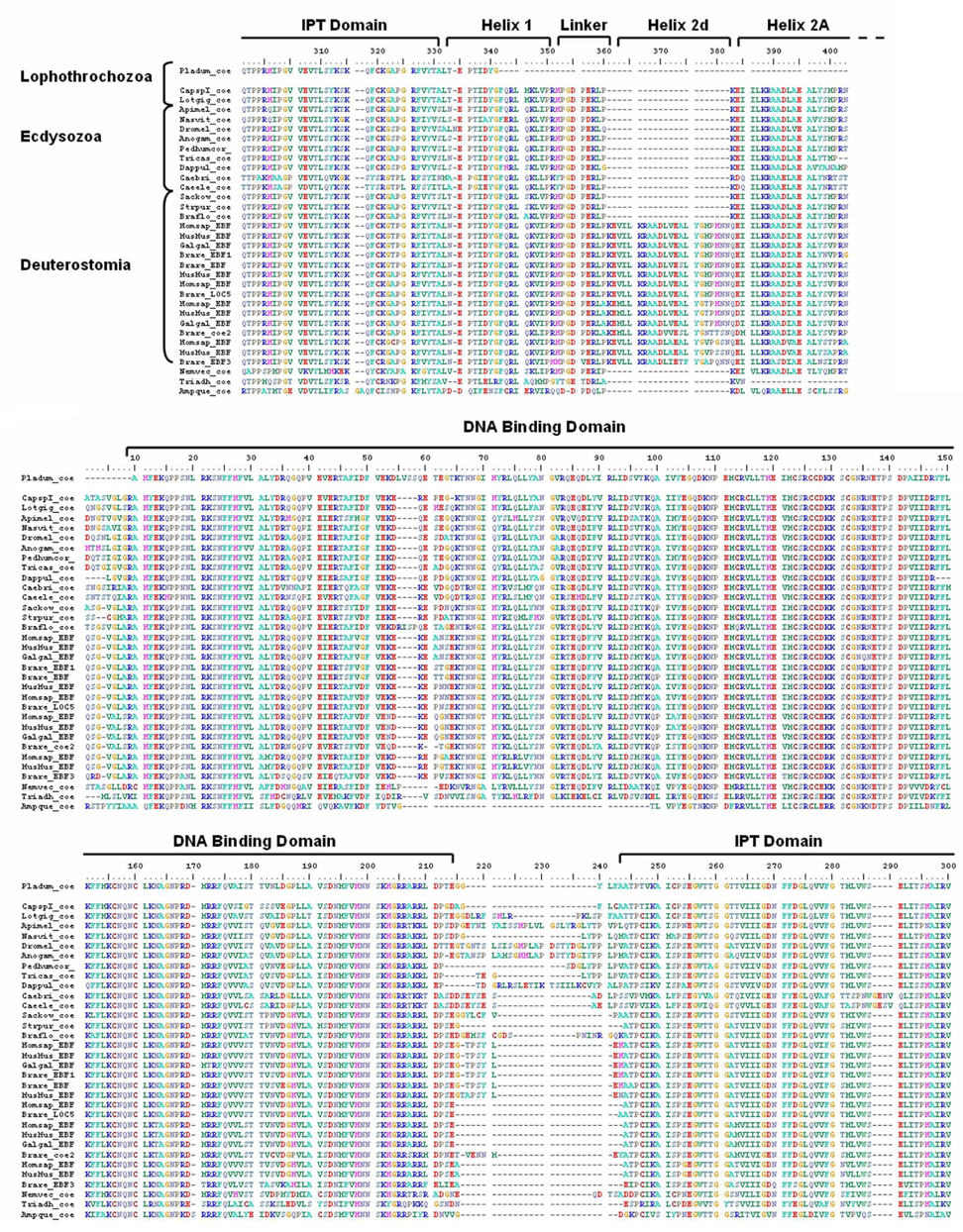

Supplement: Figure S7 — Multiple alignment used for phylogenetic analysis of COE proteins. Pladum : platynereis dumerilii ; Capspl : Capitella sp ; Lotgig : Lottia gigantea ; Apimel : Apis melifera ; Nasvit : Nasonia vitripennis ; Dromel : Drosophila melanogaster ; Anegam : Anopheles gambiae ; Pedhumcor : pediculus humanus corporis ; Tricas : Tribolium castaneum ; Dappul : Daphnia pulex ; Caebri : Caenorhabditis briggsae ; Caeele : Caenorhabditis elegans ; Sackow : Saccoglossus kowalevskii ; Strpur : Strongylocentrotus purpuratus ; Braflo : Branchiostoma floridae ; Homsap : Homo sapiens ; Musmus : Mus musculus ; Galgal : Gallus gallus ; Brare : Brachidanio rerio ; Nemvec : Nematostella vectensis ; Triadh : Trichoplax adherens ; Ampque : Amphimedon queenslandica. (TIF) [file pone.0021213.s007.tif]
